# Detection, Localisation and Assessment of Defects in Pipes Using Guided Wave Techniques: A Review

**DOI:** 10.3390/s18124470

**Published:** 2018-12-17

**Authors:** Aidin Ghavamian, Faizal Mustapha, B.T Hang Tuah Baharudin, Noorfaizal Yidris

**Affiliations:** 1 Department of Aerospace Engineering, Universiti Putra Malaysia, Serdang, Selangor 43400, Malaysia; faizalms@upm.edu.my (F.M.); nyidris@upm.edu.my (N.Y.); 2 Department of Mechanical and Manufacturing Engineering, Universiti Putra Malaysia, Serdang, Selangor 43400, Malaysia; hangtuah@upm.edu.my

**Keywords:** guided wave ultrasonic testing (GWUT), defect detection, finite element method (FEM), defect characterisations, transducer, focusing technique, reflection coefficient

## Abstract

This paper aims to provide an overview of the experimental and simulation works focused on the detection, localisation and assessment of various defects in pipes by applying fast-screening guided ultrasonic wave techniques that have been used in the oil and gas industries over the past 20 years. Major emphasis is placed on limitations, capabilities, defect detection in coated buried pipes under pressure and corrosion monitoring using different commercial guided wave (GW) systems, approaches to simulation techniques such as the finite element method (FEM), wave mode selection, excitation and collection, GW attenuation, signal processing and different types of GW transducers. The effects of defect parameters on reflection coefficients are also discussed in terms of different simulation studies and experimental verifications.

## 1. Introduction

Industrial pipeline systems are commonly used to transport oil, gas and petrochemical products (e.g., corrosive substances). In-service inspection is required to avoid catastrophic failures and to guarantee the safe operation of pipelines [1,2,3,4,5]. A defect is considered an elementary form of failure in pipes that could fail a safety system [4]. A pipeline malfunctioning due to defects could lead to a reduction in or loss of profits in the oil, gas and petrochemical industries. We refer the reader to reports on some well-known failures, such as the leaked oil pipeline of the trans-Alaska pipeline system [6] and the corroded gas pipeline in Guadalajara City in Mexico [7].

Monitoring of defects (i.e., corrosion) in inaccessible regions, such as the interface between a pipe and the pipe supports [8,9], is sometimes infeasible by conventional non-destructive testing (NDT) methods [4,10,11]. In these inaccessible areas, defects may develop rapidly and cause sudden failure [12,13]. Guided wave ultrasonic testing (GWUT) is considered a fast-screening technique to inspect long distances of a structure, such as aboveground or underground pipelines [14,15]. The specific technique for underground pipeline inspection is pigging, i.e., inspecting through inside the pipes using a unit which is equipped with an inspection system (e.g., ultrasonic or magnetic flux leakage tool) and travels along the pipeline [16]. However, significant parts of underground pipelines in electric power plants are not developed for pigging. Unpiggable pipelines can be inspected by excavating problematic sections of the pipeline from outside the pipe.

In this regard, GWUT would be helpful because details about the condition of unexcavated sections of a pipeline could be obtained around the local excavation hole [17]. Intelligent inline pigging methods can also be used to inspect oil and gas buried pipelines, but this technology is expensive and may need considerable operational interruption. In this case, the GWUT technique enables the inspection of buried pipeline sections in accessible locations from a transducer location (tool). Road crossings, especially those with underground pipes (often in a sleeve) with limited access, can be tested remotely using the guided wave (GW) technique without the need to shut them down or perform extensive excavation work [18].

Inspecting pipes in the above-mentioned situations using conventional ultrasonic methods is costly and time consuming [17,19,20,21,22,23]. The GWUT technique can also be employed to inspect insulated pipelines without the need to remove all insulation, which is an expensive process [24,25,26]. In fact, removing only a short length of insulation enables inspectors to identify defects over tens of metres under the insulation (i.e., corrosion under insulation (CUI)) [27,28]. GWUT can identify defects in structures underwater (i.e., offshore risers) [29,30], coatings [13,17,18,31,32,33,34,35] and concrete [36,37,38,39,40]. Implementing the GW ultrasonic technique allows reliable identification, defect classification, sizing of defects and reduction in the overall inspection cost [41,42,43,44,45,46,47].

**Figure 1:**
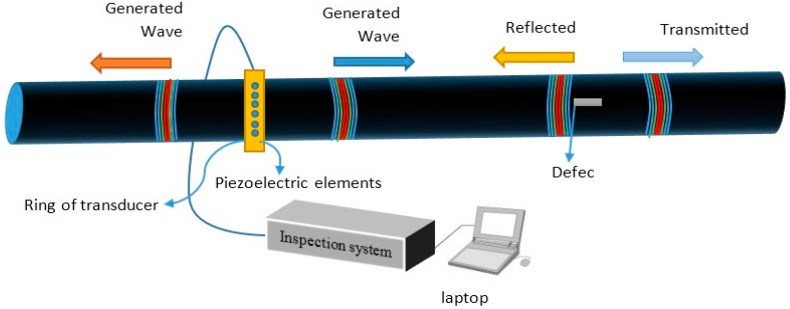
Pipe inspection system using GW.

The GWUT technique has been commercially available for over 20 years for corrosion monitoring in the oil, gas and petrochemical industries [31,48,49,50], and it is a suitable inspection technique for pipes measuring 2–48 inches in diameter. The GW test system uses a pulse-echo method to generate and receive waves by employing rings of transducers that comprise piezoelectric elements embedded at a single transducer location surrounding the pipe circumference (Figure 1). Once the elements are excited equally and simultaneously, an axisymmetric mode is generated and travels along the pipe wall [2,19,24,26]. The locations of internal and external defects in the pipe wall are identified by their reflection arrival time. In contrast to bulk waves, guided waves (GWs) are bounded by the entire wall of the pipe through which they can propagate along the pipe length. Partial reflection occurs when the waves impinge on any features that locally change the pipe geometry [51,52,53], such as pipe welds [11,54] and defects in the form of corrosion or cracks [38,54,55,56,57,58,59,60]. Nevertheless, difficulties arise from the irregular shapes of corrosion defects and the complexity of GWs which makes quantitative measurements challenging.

As pioneers, Rayleigh in 1887 and Lamb in 1917 [61] investigated the propagation of stress waves in isotropic 2D elastic plates with free-boundary conditions. During the last century, GW theory has been broadly studied by scholars such as Thomson in 1950 [62], Gazis in 1959 [63], Victorov in 1970 [64] and Auld in 1990 [65]. A comprehensive analysis of GWs was presented by Rose in 1999 [66]. Experiments on GW-based inspection were performed by Alleyne et al. in 1998 [26] and Cawley et al. in 2003 [67]. The current study reviews the detection, location and assessment of various defects in industrial pipes as waveguides based on guided ultrasonic wave (GUW) techniques. The limitations and capabilities of the GW techniques for corrosion monitoring using, simulation techniques, commercial GW systems and GW focusing techniques are discussed. 

The effects of defect parameters on reflection coefficient (RC) according to the findings of extensive theoretical and experimental studies are also reviewed. The attenuation and effects caused by pipe features, pipe surface condition, surrounding materials and defect detection in buried pipes under pressure are discussed. A review of defect identification in pipes based on GW techniques is presented in Table 1.

## 2. GWs’ Properties

Acquiring previous knowledge on propagating medium, the physical characteristics of the pipe, the governing equations for GW propagation in pipes, guide wave dispersive behaviour, dispersion curves, mode shapes, pure mode selection and different types of defects that may cause GW reflection is important [86]. As a prerequisite, such knowledge helps in the identification of reflected waves using particular signal processing techniques. Many techniques have been developed to determine the features and characteristics of reflected waves. The results of the analyses reveal the existence, location and extent of defects [1,2,26,70,77,87,88]. 

### 2.1. Governing Equation for GW Propagation Using Cylindrical Coordinates

Provided that traction-free boundary conditions are considered for an elastic isotropic hollow cylinder surface, Navier’s governing displacement equation regarding GWs can be written as:

(1) μ∇2U→+(λ+µ)∇∇.U→=ρ(∂2→U∂t2)

where t is the time; $\vec{U}$ denotes the displacement vector; ρ represents the density; $\nabla^{2}$ indicates the Laplace operator; µ and *λ* are Lame’s constants; ρ, *λ* and µ determine the velocities of the bulk waves in the material. If $C_{2}$ is the velocity of the shear (SH) and $C_{1}$ represents the dilatational velocity of the longitudinal bulk wave, then:

(2) $${\;C}_{1}=\;\sqrt{\frac{\lambda +2\mu }{\rho }},$$

(3) $$C_{2}=\;\sqrt{\frac{µ}{\rho }}.$$

The investigation of wave propagation in hollow cylinders was conducted by Gazis in 1959 [63]. The cylinder was regarded as isotropic, and he decomposed Navier’s equation using the Helmholtz decomposition technique to simplify the problem into:

(4) $$\vec{U}=\;\nabla \Phi +\nabla \times \vec{H}$$

The equivoluminal vector potential $\vec{H}$ and the dilatational scalar potential Φ were used to explain the displacement field. Boundary conditions are required to address the governing equations. Traction-free boundary conditions exist for the outer and inner surfaces of a hollow cylinder. A hollow cylinder is assumed to be infinitely extended, and the boundary conditions can be simplified by defining gauge invariance as:

(5) $$\nabla .\vec{H}=0$$

Here, the gauge invariance conditions are considered the equal volume conditions (a structure at constant volume). The gauge invariance can be applicable due to the infinite volume and length of the hollow cylinder. The boundary conditions at both ends of the hollow cylinder need not be considered using gauge invariance conditions.

Substituting Equation (4) into Equation (1) gives:

(6) $$\nabla^{2}\Phi =\;\frac{1}{C_{1}}\frac{\partial^{2}\Phi }{\partial t^{2}}$$

(7) $$\nabla^{2}\vec{H}=\;\frac{1}{C_{2}}\frac{\partial^{2}\vec{H}}{\partial t^{2}}$$

In cylindrical coordinates, the potentials Φ and $\vec{H}$ have subsequent forms depending on elasticity theory, namely:

(8) $$\nabla^{2}\Phi =\;\frac{\partial^{2}\Phi }{\partial r^{2}}+\frac{1}{r}\frac{\partial \Phi }{\partial r}+\frac{1}{r^{2}}\frac{\partial^{2}\Phi }{\partial \theta^{2}}+\frac{\partial^{2}\Phi }{\partial z^{2}}$$

(9) $$\vec{H}=H_{r}{\vec{e}}_{r}+H_{\theta }{\vec{e}}_{\theta }+H_{z}{\vec{e}}_{z},$$

(10) $$\begin{array}{c} \nabla^{2}\vec{H}=\nabla^{2}\left(H_{r}{\vec{e}}_{r}+H_{\theta }{\vec{e}}_{\theta }+H_{z}{\vec{e}}_{z}\right)\\ =\;\left(\nabla^{2}H_{r}-\;\frac{1}{r^{2}}H_{r}-2\frac{1}{r^{2}}\frac{\partial H_{\theta }}{\partial \theta }\right){\vec{e}}_{r}+\left(\nabla^{2}H_{\theta }-\;\frac{1}{r^{2}}H_{\theta }+2\frac{1}{r^{2}}\frac{\partial H_{r}}{\partial \theta }\right){\vec{e}}_{\theta }+\nabla^{2}H_{z}{\vec{e}}_{z}. \end{array}$$

The expression of the potentials was proposed by Gazis [63] to split the variables, i.e.,:

(11) $$\begin{array}{cc} \begin{array}{l} \Phi =f\left(r\right)\Theta \left(n\theta \right)e^{i\left(\mathrm{kz}-\omega t\right)}\\ H_{r}=h_{r}\left(r\right)\Theta_{r}\left(n\theta \right)e^{i\left(\mathrm{kz}-\omega t\right)}\\ {\;H}_{\theta }=h_{\theta }\left(r\right)\Theta_{\theta }\left(n\theta \right)e^{i\left(\mathrm{kz}-\omega t\right)}\\ {\;H}_{z}=h_{z}\left(r\right)\Theta_{z}\left(n\theta \right)e^{i\left(\mathrm{kz}-\omega t\right)} \end{array} & \left(n\;=\;0,\;1,\;2,\;\ldots \right) \end{array}$$

The circumferential order of the ‘a’ wave mode is called the integer n, and the wavenumber is known as K. The unknown coefficients are $h_{\xi }$(r) (ξ = r,θ,z) and f(r). In case the continuity conditions are considered in relation to θ and θ+2π, the functions $\Theta_{\xi }\left(n\theta \right)$ (ξ = r,θ,z) and Θ(nθ) must just include cos(nθ) and/or sin(nθ). Thus, the next assumption was presented by Gazis as:

(12) $$\begin{array}{cc} \begin{array}{l} \Phi =f\left(r\right){\cos n\theta e}^{i\left(\mathrm{kz}-\omega t\right)}\\ H_{r}=h_{r}\left(r\right)\sin{n\theta e}^{i\left(\mathrm{kz}-\omega t\right)}\\ H_{\theta }=h_{\theta }\left(r\right)\cos{n\theta e}^{i\left(\mathrm{kz}-\omega t\right)}\\ H_{z}=h_{z}\left(r\right)\sin{n\theta e}^{i\left(\mathrm{kz}-\omega t\right)} \end{array} & \left(n\;=\;0,\;1,\;2,\;\ldots \right) \end{array}$$

Equation (12) expresses the displacement potentials as:

(13) $$\begin{array}{l} U_{r}=A_{r}\left(r\right)\cos{n\theta e}^{i\left(\mathrm{kz}-\omega t\right)}\\ U_{\theta }=A_{\theta }\left(r\right)\sin{n\theta e}^{i\left(\mathrm{kz}-\omega t\right)}\\ \;U_{z}=A_{z}\left(r\right)\cos{n\theta e}^{i\left(\mathrm{kz}-\omega t\right)} \end{array}$$

According to Equation (13), in case of axisymmetric modes, n = 0, $U_{\theta }\;$= 0 can be observed. However, the axisymmetric torsional modes have a dominant displacement in the circumferential direction ($U_{\theta })$; consequently, Equation (12) cannot be considered as a proper assumption for the torsional modes. Only calculations of longitudinal modes are therefore accurate according to Gazis’ solutions. Nevertheless, some scientists have stated that torsional and longitudinal waves can be expressed by Gazis’ solutions. In 2005, alternate solutions were presented by Rose, Sun and Zhang for torsional modes [20,51,89,90,91]:

(14) $$\begin{array}{cc} \begin{array}{l} \Phi =f\left(r\right)\sin{n\theta e}^{i\left(\mathrm{kz}-\omega t\right)}\\ H_{r}=h_{r}\left(r\right)\cos{n\theta e}^{i\left(\mathrm{kz}-\omega t\right)}\\ H_{\theta }=h_{\theta }\left(r\right)\sin{n\theta e}^{i\left(\mathrm{kz}-\omega t\right)}\\ H_{z}=h_{z}\left(r\right)\cos{n\theta e}^{i\left(\mathrm{kz}-\omega t\right)} \end{array} & \left(n\;=\;0,\;1,\;2,\;\ldots \right) \end{array}$$

The simplified case of Equation (14) can be represented below only when axisymmetric wave modes are considered:

(15) $$\begin{array}{c} \Phi \;=\;f\left(r\right)e^{i\left(\mathrm{kz}-\omega t\right)}\\ H_{r}=h\left(r\right)e^{i\left(\mathrm{kz}-\omega t\right)} \end{array}$$

On the basis of the above equations and linear elasticity theory, the dispersion equation for harmonic waves in infinite harmonic hollow cylinders can be expressed as the following eigenvalue equation:

(16) $${\left|C_{ij}\right|}_{8\times 8}=0,\;\mathrm{of}\;\mathrm{which}\;K_{1}\;=\;\frac{\omega }{C_{1}}\;\mathrm{and}\;K_{2}\;=\;\frac{\omega }{C_{2}}$$

where K is the wave number function, ω is the angle frequency and C_ij_ is the diameter [20,51,89,90,91]. The above eigenvalues result in dispersion curves that can be fit to hollow cylinders.

### 2.2. Dispersion Curves and Mode Shapes

Despite the promising capabilities of GWs in inspection applications, inherent difficulties related to their dispersive nature and multimode propagation exist [92]. The standard BS9690 for GW testing indicates that frequency is independent of the GW’s acoustic properties, such as attenuation and velocity [93]. In this framework, the dispersion curve shows group and phase velocities at a particular frequency [94]. Dispersion curves can graphically display the dependence of velocity on frequency. Wave mode velocities and mode shapes are functions of frequency which cause them to be ‘dispersive’ [95]. *Phase velocity* is the name given for this calculated velocity. The phase velocity of flexural and longitudinal modes depends on wall thickness and pipe diameter [51]. If the circumferential order increases, then the phase velocity increases. The concept of *group velocity* must be noted as wave energy propagates with this velocity from one point of solid media to another point [20,24,51,67,96]. In fact, a finite pulse is supposed to be generated by a time-dependent force *P*(*t*). A superposition of many waves of distinctive frequencies is accordingly considered as this finite pulse. Each wave is assumed to exist within the zone of −∞ < x < +∞. Indeed, a finite pulse is generated when all waves are added. 

**Figure 2:**
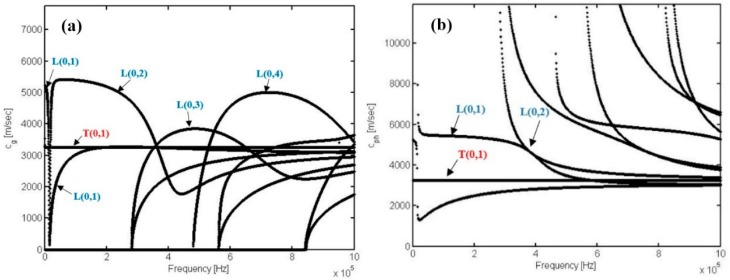
Sample of (**a**) group velocity dispersion curve and (**b**) phased velocity dispersion curve for a 6.5-inch outer diameter (OD) schedule 40 steel pipe with a thickness of 6 mm, including all of the torsional mode T(0,m) and longitudinal mode L(0,m), i.e., (m = 1,2,3,…), and non-axisymmetric modes of all T(n,m) and L(n,m), i.e., (n = 1,2,3,…,m = 1,2,3,…) [99].

The above-mentioned concept is the same as the concept of Fourier series summation. Therefore, a group of waves as pulse A propagate with a group velocity (C_g_). After passing time t, the entire pulse propagates (at a distance of t × C_g_) to point end [97]. The dispersion effect is characterised by the propagation of wave packet energy (like pulse A) at different velocities depending on its frequency content [98]. Dispersion curves actually play a key role in GW non-destructive evaluation (NDE) [51]. The possibility of mode excitation can be determined with dispersion curves [96]. In addition, the simulation of dispersive GW propagation can also be realized by using dispersion curves and any indicated frequency spectrum [51]. Figure 2a,b shows a sample of phased velocity and group velocity dispersion curves and the wave structures of a 6-inch schedule 40 steel pipe. The curves were measured by the dispersion GUIGUW software which was supported by the *GUIGUW* team [99]. 

### 2.3. Mode Types and Nomenclature

Meitzler in 1961 [100] proposed a classification which divided GW modes into three basic categories, namely, (1) T(0,m) representing axisymmetric torsional (T) modes, (2) L(0,m) representing axisymmetric longitudinal (L) modes (the second integer for both modes is m = 1, 2, 3, 4, …) and (3) F(n,m) representing non-axisymmetric flexural (F) modes (the first and second integers are n = 1, 2, 3, 4, …, m = 1, 2, 3, 4, …, respectively). Waves can follow a curved path and propagate in the circumferential direction. The first integer ‘n’ shows the harmonic variation order that involves stresses and the resulting displacements around the pipe circumference. The family of modes is shown with the second integer ‘m’ as a counter variable [51,60,101,102]. 

**Figure 3:**
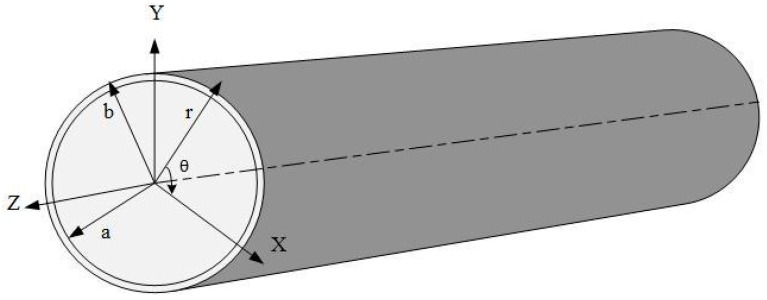
System of cylindrical coordinates (r,θ,z) for a cylindrical waveguide.

The torsional and longitudinal modes are axisymmetric when the first integer is zero. The dominant particle motion of the torsional mode is in the θ direction (circumferential particle motions which are perpendicular to the wave direction, as depicted in Figure 3), whereas the dominant particle motion of the longitudinal mode is in z and/or r orientations parallel to the wave direction (axial or radial orientations and no motion in the circumferential direction, as shown in Figure 3). The L(0,2) mode has minimal radial energy leakage, and its radial displacement is relatively smaller than the axial displacement. Hence, this mode can propagate over a long distance and is consistent with the long-range requirements of GW testing. This mode also significantly enhances the efficiency of defect detection. T(0,1), on the contrary, is highly sensitive to circular, axial, external and internal defects. The flexural GWs include non-axisymmetric and axisymmetric modes in accordance with the distribution of the wave energy along the circumferential direction [51,100,103]. All three components of displacement, namely, circumferential, axial and radial (r, θ, z), exist for flexural waves, as demonstrated in Figure 3 [66,104]. 

In-line inspection or corrosion monitoring in a pipe can be performed at any angle of the pipe circumference which is under the coverage of district transducer arrays [51,105]. However, only a part of the pipe circumference can be covered by acoustic fields of non-axisymmetric flexural modes (F(n,m)) [15,24,106]. For each circumferential order ‘n’, including both torsional and longitudinal modes, an unlimited number of modes exist (Figure 2a,b) [96]. In this case, one or two cycles of stress and displacement variation occur around the circumference for all modes of order one and order two, respectively [107]. The acoustic fields of axisymmetric modes according to the structural health monitoring (SHM) system for hollow cylinders, such as pipes, were presented in the work of Kang et al. [15]. Eight piezo-composite transducer arrays with equal circumferential spacing were mounted on an ASTM A106 6-inch schedule 40 carbon steel pipe to cover the entire pipe circumference by an acoustic field of axisymmetric modes (L(0,m)).We refer the reader to Section 3 for additional details.

### 2.4. Pure GW Mode Selection

Many modes exist at a particular frequency. They are so close that they can possibly propagate simultaneously and thereby make the received signals too complicated for analysis. Hence, pure mode generation is difficult at the given frequency range. However, when the selected mode is dispersive, the GW energy spreads out in time and space, thereby reducing the amplitude so that the signal is lost in the noise. In other words, various components of frequency that travel at various velocities can increase the signal duration and thus compromise the resolution [20]. Although each parameter used in the GWUT is frequency dependent, signals can be reliably interpreted, and some of the dispersive effects can be negated through the correct design of transducer arrays, signal processing, instrumentation and aid from an operator with inspection experience [22]. Therefore, mode selection is affected by the ease of mode generation whilst preventing the generation of other modes. Substantial efforts have been focused on the excitation of a single mode to improve the sensitivity to different defects that change the mechanical impedance of a structure. The dispersion problem can be controlled by generating a narrow frequency band focused on an area where the mode of interest is non-dispersive. Since the excitation frequency may be close to the resonance frequency of the transducers, a narrow-band frequency signal is required to avoid signal distortion. Generally, narrow band signals are modulated in a 5-cycle or 10-cycle Hann-window or an 8–10-cycle Gaussian window (refer to Section 4 for more details) [52,92,96,98,108,109,110]. 

The generation and reception of unwanted modes lead to an increase in coherent noise and subsequently reduce the signal to the coherent noise ratio; this condition exerts an effect on the sensitivity of a test [34,51,96,111]. However, if the arrays are designed correctly, multimodal GW signals that propagate in a pipe can be separated into their component wave modes by using a spatial filtering technique. Thus, each mode can be processed or analysed separately whilst providing information about the angular orientation and phase of the selected mode. At a determined angular orientation, this technique can selectively excite a selected wave mode and measure the relative amplitude of the selected mode that is presented in a specimen [34,51,96,111]. The signal-to-noise ratio (SNR) can be modified by employing a spatial filtering technique and the correct recombination of the signal. In fact, the shape of the selected filter can match that of the displacement distribution of a specific mode. The received signals at spots on the circumference are multiplied by a scaling factor determined by the related shape of the filter. This provides the result of eliminating all but the targeted mode from the multi-mode signal. This technique can be used for potential novel defect sizing algorithms and advanced signal processing technique [112,113]. 

## 3. Guided Waves (GWs) Interaction with Defects

GWs exhibit continuous interaction with waveguide boundaries whilst propagating across long distances. In addition, upon encountering an anomaly or defect, GWs interact with the defect, which leads to reflection. However, interference occurs between the reflected signals scattered by the defect and those received from the pipe welds. Dispersion and mode conversion also occur whilst GWs interact with notches, thereby causing their complexity [114]. In general, the interaction of waves with a damage (e.g., a nonsymmetric defect that causes leakage) results in refraction, reflection and the conversion of energy amongst different modes that further complicate signal processing but can be predicted by using suitable boundary conditions [115,116,117]. Hence, the interaction of guided ultrasonic waves with defects in structures is a complex physical phenomenon that should be understood as fundamental information before simulating GWs or any actual usage of the GW technique [118]. 

Discontinuities in a structure can either be discontinuities caused by material property variations, including a structure that is partly embedded into a surrounding medium (refer to Section 7.1 and Section 7.2 for additional details) and two different materials welded together, or geometric discontinuities, which include course corrosion defects, free ends, curved parts attached to the main structure and welds that connect two parts [118]. 

**Figure 4:**
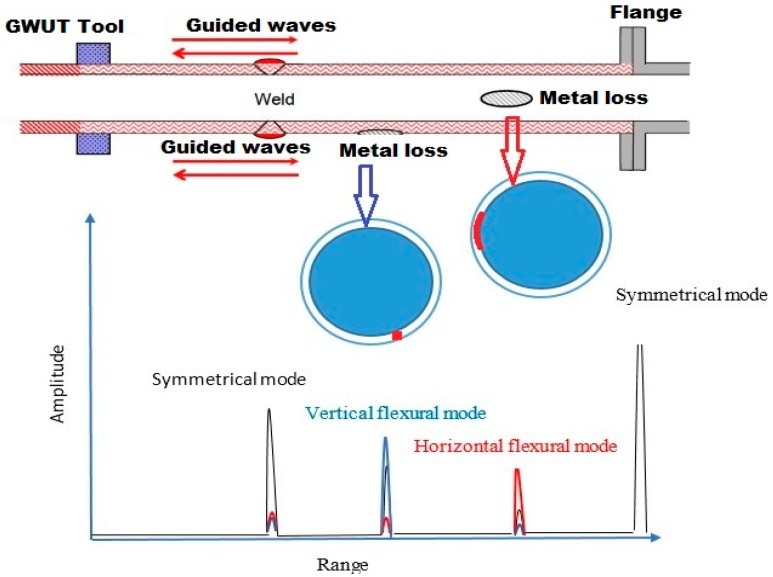
Propagation and reflection of GW in a pipe. The axisymmetric and non-axisymmetric horizontal or vertical flexural modes reflected from symmetric and non-axisymmetric pipe features, respectively.

Direct reflection occurs if GWs interact with pipe features. Three series of time-dependent data can be typically collected for further signal processing: (1) vertically arranged flexural mode, (2) horizontally arranged flexural mode and (3) symmetric mode (Figure 4). Low et al. [22] and Demma et al. [24], indicated that only axisymmetric modes are reflected provided that these modes are considered incident modes acting on axisymmetric pipe features, such as flanges, uniform welds (with weld caps) and square ends. Axisymmetric reflections from pipe features (e.g., flange and uniform welds) are shown in Figure 4. However, according to Lowe et al. [2,22], Carandente et al. [74] (only the reflection of T(0,1) was presented) and Li et al. [119], if the features are non-axisymmetric, such as localised corrosion patches on a pipe, then some of the non-axisymmetric waves can be recognised because of the mode conversion phenomenon in flexural modes, as shown in Figure 4 (refer to Section 3.1 for additional details). Moreover, non-axisymmetric features (i.e., corrosion) should be distinguished from axisymmetric features (i.e., flanges, welds and square ends) [24]. Current commercial GW systems can distinguish vertically and horizontally flexural modes from axisymmetric modes by comparing the received signals from the segments of the transducer array. The amplitude of each time-dependent series can be assessed such that feature types can be drawn as conclusions (refer to Section 6 for additional details). 

### 3.1. Effect of Geometric Parameters on the Reflection Coefficient

**Figure 5:**
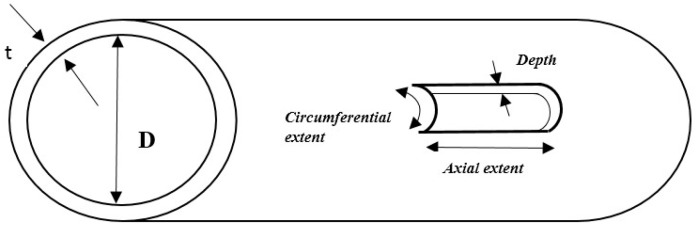
Three-dimensional defect parameters (radial depth, axial extent, and circumferential extent); D: pipe diameter; t: thickness.

The RC can be determined basically by dividing the reflected mode amplitude from the defect by the reference mode amplitude obtained from the pipe end before introducing the defect. The effects of the geometric parameters of a defect on the RC can be identified and quantified to characterise a pipe defect [72]. Generally, the RC depends on particular parameters that can be used for a quantitative study. The parameters include (1) modes that are excited, (2) frequency (f) of the excited modes, (3) pipe thickness (t), (4) pipe size (diameter; D), (5) circumferential extent of defect (c), (6) axial extent of defect (a) and (7) defect depth (b). Figure 5 shows the last three parameters with regard to a pipe [24,26,118,120,121,122].

Several studies have indicated that the *circumferential extent* of a defect and the defect depth are determined as variable parameters that can control defect reflections. A number of studies have also discussed the influence of the circumferential extent of defects on the reflection from a notch using incident modes, such as L(0,2) [2,26,72,123] and T(0,1) [56], at high frequency–diameter (fd) products (more than approximately 3000 kHz mm). Such studies have observed that the RC shows a linear behaviour with the circumferential extent of non-axisymmetric defects (i.e., non-axisymmetric cracks [56]) at proportionately high frequencies; meanwhile, at low frequencies, this behaviour becomes ‘concave’ and small, particularly for low-defect circumferential extents [56,123]. In the work of Alleyne et al. [26], a linear behaviour was obtained for a reflection amplitude of the L(0,2) incident mode from a notch of varying circumferential extents at 70 kHz and a half wall thickness in a 3 inch pipe. Notably, the advantages of using the L(0,2) incident mode within the frequency range of 50 kHz to 100 kHz include its nondispersive behaviour and ability to provide 100% coverage for the pipe wall [15]. In the same work presented by Demma et al. [24], a linear behaviour was observed for the RC of L(0,2) and T(0,1) as axisymmetric modes from a defect circumferential extent only if the examination was conducted approximately in the high-frequency range. The authors indicated that the reflected signal from a defect with 100% depth (through-thickness) was roughly considered independently from frequency. Furthermore, the defect circumferential extent at 100% depth influenced the RC of the axisymmetric incident L(0,2) mode that was converted into F(1,3). This phenomenon could also be observed for T(0,1) as an incident mode that was converted into F(1,2) [24]. As noted in Ref. [2], mode conversion into flexural F(1,3) modes as a non-axisymmetric mode can be distinguished from the L(0,2) mode. Nevertheless, given the existence of defects with a small circumferential extent, the ratio of the reflection component of mode (F) to the reflection component of mode (A) cannot be adequately sensitive to small changes in the circumferential extent of torsional and longitudinal modes [14]. In other words, whether defects extend over the circumference (i.e., 5% or 10% of the circumference) cannot be easily specified [14]. Therefore, these flexural modes are aligned in any orientation during propagation in a pipe such that in practical tests, the localised metal loss features, such as corrosion, become consistent with the circumferential orientation of flexural modes causing the reflection [95].

With regard to the dependence on the axial extent of a notch, the interaction phenomenon due to the reflection from the back and front edges of a notch results in periodic variations in the RC [56]. The effects of the geometric parameters of a defect on the two reflected signals from the back and front defect edges indicate different signal features, leading to the complication of the entire reflected signal [72]. In 2002, Cawley et al. [123] published a paper in which they explained that in a part-thickness notch, such as an axisymmetric crack of a minimal axial extent of a given circumferential extent with respect to the RC, an increasing trend could be observed from the notch with frequency at a provided depth. Behaviour monotonically increasing with depth could also be recognised at all frequencies. In another work, Demma et al. [56] proposed that when the incident mode T(0,1) propagates in a 24 inch pipe at 35 kHz with an axisymmetric part-depth notch (b = 0.5t, 50% depth) of varying axial extents, the RC alters cylindrically (cylindrical behaviour) with the axial extent of the notch. The maximum occurs when the wavelength reaches 75% and the notch width reaches 25%. These interference effects occur for square-sided defects and become less severe in the case of real defects. Cawley et al. [123] showed that this cylindrical behaviour occurs when the wavelength in comparison with the thickness of the pipe wall is long. In other words, this behaviour is observed when the ratio of the pipe wall thickness to the wavelength is 10% or less. Cawley and colleagues conducted an analysis of a 3 inch pipe with modes L(0,1) and L(0,2) at 5, 40 and 80 kHz, as well as a 24 inch pipe at 10 kHz; the RC were examined for varying defect axial extents with a part-depth defect (b = 0.2t, 20% depth) for an axisymmetric notch. Demma et al. [24] observed a dependency between the axial extent of a defect and the wavelength of the excited mode related to the RC from defects, including the minimum and maximum values at which the axial extent reached 5% and 25% of the wavelength, respectively. As mentioned previously, the GW reflection from defects is the joint consequence of the interference between two signals reflected from the back and front edges with different signal features (a resonance phenomenon). This interference results in the previously mentioned cylindrical behaviour. Detailed information about the cause of this cylindrical behaviour is available in the works of Tse et al. [124] and Wang et al. [72]. Demma et al. [125] determined that depending on the axial extent of a notch and excitation frequency, the back and front edge reflections of a notch can interfere destructively or constructively. No reflection exists when a notch’s axial extent is equal to the wavelength ratio of 45%; in this case, the notch would not be detected. However, in practice, an excitation signal is introduced in a Gaussian window tone burst so that its energy distributes over a range of frequencies [24]. As a case in point, for a windowed tone burst including five cycles with a −6 dB bandwidth and approximately 30% of centre frequency, the defect’s axial extent is 45% of the wavelength because the availability of other frequencies in the reflected signals from the notch is recognised even at the centre frequency [24,56,123]. Furthermore, Lowe et al. [22], Demma et al. [24,56], Cawley et al. [123] and Alleyne et al. [26] showed that a peak can be detected at a defect extent of approximately 25% of the wavelength. The interesting part is that the peak of the RC for defects including low axial extents, such as cracks, is less than that for defects, such as corrosion patches, when the axial extent makes up a significant portion of the wavelength and can therefore be observed to be less complicated than cracks. This finding indicates that GWs are more sensitive to corrosion patches than cracks in the range of low test frequencies. In fact, this finding can be attributed to the large reflection of summed in-phase scattered waves from a rough corrosion patch having more successive steps than that when more phase cancellations occur at high frequencies. Therefore, the influence of the axial extent of a defect is relevant for corrosion patches as a defect (metal loss over a significant pipe length) that influences reflectivity. Regarding the previously mentioned studies, a similar behaviour can be observed for incident axisymmetric modes, including L(0,1), T(0,1) and L(0,2), which propagate over pipes of all sizes, defect depths and frequencies [22,24,26,56,123]. Thus, we can conclude that testing a defect of a particular axial extent at more than one frequency is inevitable because of destructive interference which causes the apparent loss of defects [56].

## 4. GW Simulation

For either single-layer or multi-layer straight pipes, theoretical methods of GWs have been used to deal with the problem of wave propagation. However, analytical solutions are difficult to obtain, and they are unavailable in many cases, e.g., the propagation of GWs in pipes with complicated shapes (including some complex features, such as welds or elbows, or defects with irregular shapes) and the propagation of GWs in anisotropic or inhomogeneous media. Some approximation methods need to be adopted to computationally evaluate the theoretical and analytical expressions of wave scattering coefficients [71]. The numerical analysis of the propagation of GWs in difficult cases can possibly lead to a final solution due to the rapid developments in computer technology over the last four decades. Four types of numerical methods are generally employed because of the difficulties and complications in determining analytical solutions. These methods, including boundary element method (BEM), finite difference method (FDM), semi-analytical finite element (SAFE) method and finite element method (FEM), have accordingly been developed to predict GW characteristics and properties in elongated structures such as pipes [86].

In this study, emphasis is placed on the application of FEM. FEM is an appropriate numerical approach for finding a solution to wave scattering problems due to complicated geometries or defects. The FEM simulation of GW propagation deals with problems caused by wave interactions with defects in structures such as pipes [5,56,71,77,123]. In this regard, the 3D model of a structure is divided into smaller elements by FEs [88,126]. FEM partitions comprise many FEs, and the element size depends on the wavelength of the propagating wave. A governing equation is assigned to each element as an individual unit having a rather simple shape. The GW propagation problem with several numbers of elements can be analysed by employing high-performance computers [86]. FEM solutions include two main types, namely, *implicit* and *explicit* analyses, which can be used to deal with various problems. Implicit analysis can solve nonlinear problems due to convergent solutions for each state received through iteration. Explicit analysis is usually used for problems of linear wave propagation along a structure as every kinematical state is calculated through the previous state [1,86,127,128,129,130,131]. For models including phased array sensors, force loadings can be used instead of displacement loadings. In several cases, including partial loading (to be used in the GW phased array focusing models), the loaded area is spatially calculated with a cosine function, such that the edge of the loaded region has zero load and the centre of the loaded region provides the maximum amplitude. Hence, unwanted modes, such as L(m,2), can be reduced using a weighting scheme [127].

The numerical analysis of GW interactions with individual defects requires the implementation of 3D solid models [2,26,56,123,125]. Moreau et al. [83] investigated the 3D scattering of GWs from clusters and irregularly shaped defects in plates using FE methods. Nevertheless, 3D models are expensive computationally [2,24]. In studying a particular 3D model and reducing mathematical problems [132], 3D models can be combined with 2D models [118]. Zuo [5] performed 2D and 3D FE analyses of the reflections by axisymmetric and non-axisymmetric defects, such as corrosion. A 2D axisymmetric moulding as a precalculation of incident fields was applied at the cross-sectional area (CSA) of a hollow cylinder under the given boundary conditions of the results in the reflection calculation of various defects. This technique decreased the computation time and the total elements needed with respect to 3D models. In sum, GW reflection behaviour from various axisymmetric and non-axisymmetric defects can be analysed by using 2D and 3D FE models which are categorised as [123] (1) axisymmetric FE models, (2) membrane FE models and (3) 3D FE models.

Axisymmetric defects which remove parts of the wall thickness and extend over the pipe circumference can be analysed by applying a 2D axisymmetric FE model. For instance, Alleyne et al. [26] conducted a 2D axisymmetric analysis of cracks by disconnecting neighbouring elements. Defects which include a finite axial extent or a varying axial extent were analysed by removing the mesh elements [56,123]. In 2002, Zhu [5] used a 2D axisymmetric FE model with 20-node brick elements (with one element covering the wall thickness), a circumferential length with 80 elements and a surrounding half-circle circumference with 12 elements to pre-calculate the incident mode L(0,2) at 100 kHz and a CS of a hollow cylinder. The results were used as a prescribed boundary condition for different axisymmetric and non-axisymmetric corrosion defects in the reflection calculations. The computation time and the number of elements needed in the 3D modelling were considerably reduced. This type of model involves 2D [123] or 3D [118] analysis depending on the elastic wave propagation in a 2D or 3D structural membrane [133].

Membrane elements for pipes discretise pipe geometry (i.e., pipe wall) [56,134]. Membrane elements can be used for defects of varying axial extents, including removing a portion of the full wall thickness of the pipe circumference [123]. Alleyne et al. [26] conducted quantitative research on the L(0,2) mode reflection from pipe notches and determined that membrane elements provide a simple alternative to solid models with constant axial displacement and nearly linear radial displacement variations through the wall thickness. The relationship between the RC of a through-thickness notch and its circumferential extent has been investigated using the membrane element model. In 1998, Low et al. [2] published their study in which they employed “membrane” elements in the FE model. Hence, their analysis was limited to through-wall (100% through-wall thickness) defects. In 2003, Demma et al. [56] conducted the same investigation in which membrane elements and the results of an axisymmetric model were combined to predict the particular effects related to various through-wall depths and the circumferential extents of the notch [112].

Several investigations into 3D solid models [63,88,135,136] have analysed the interaction of GWs with individual defects [118]. Any stress direction and displacement in 3D models are allowed. A 3D model is used to examine all types of axisymmetric or non-axisymmetric defects to ensure that the full wall thickness or even a portion of the pipe circumference can be removed. However, more through-wall thickness (e.g., 100% thickness) elements that are less computationally expensive are needed to conduct an investigation into the influence of defect depth [24]. In 2003, Demma et al. [56] published a paper that reported their use of solid brick elements as through-thickness elements. However, as the goal was to develop a model that was computationally cost effective, they modelled a pipe that included a combination of a part-thickness, part-circumference notch and an axisymmetric part-depth notch such that it could be conducive to predicting the reflection from part-circumference and part-thickness defects over a large frequency range. In several cases, a 3D analysis of the full length of a pipe, including the previously mentioned notches at some distance along it, would be possible. However, as already mentioned, a shortened pipe length would be adequate to reduce the computation time [2,137,138]. The signal of an input wave is excited through the model by prescribing time-varying displacements at one end of a pipe. 

### 4.1. Excitation and Signal Processing

Either a displacement or force loading can be applied to a subsection of the model surface to illustrate the loading function. Most FE modelling was conducted by employing displacement loading because of the consistency of its results relative to those of force loading [139]. Mode cancellation cannot be used with displacement loading. This limitation is considered a drawback of this type of loading. If two rings of transducers are employed with phase delays between them, then the first transducer ring to be excited causes the generation of a bidirectional mode. The second transducer ring excites a new bidirectional mode or makes a clamped boundary condition on the basis of the second ring loading. An experimental system is not simulated by either case [126,140,141]. The source (excitation nodes as transducers) can be arranged at the end of the model so that the model excludes a backward direction to enable mode control of the energy propagation in the backward direction. Moreover, the energy along one direction can be dissipated by applying viscous damping. When applying individual and non-phased transducers, a good choice for simulations is a displacement boundary condition [142,143,144]. However, several loading steps must be created when applying phased array transducers with displacement boundary conditions, and the calculations of the displacement amplitude to be made for each transducer through each step may become difficult to perform and apply [145,146,147]. By contrast, force loadings do not need any particular requirement after the loads have been applied if the last value in the force load is zero. The wave propagation is not affected after the loads have been applied when imposing a traction-free boundary. Moreover, force loadings can be used to perform mode cancellation or counteract the propagation of the wave in several directions without using a clamped boundary [127]. 

**Figure 6:**
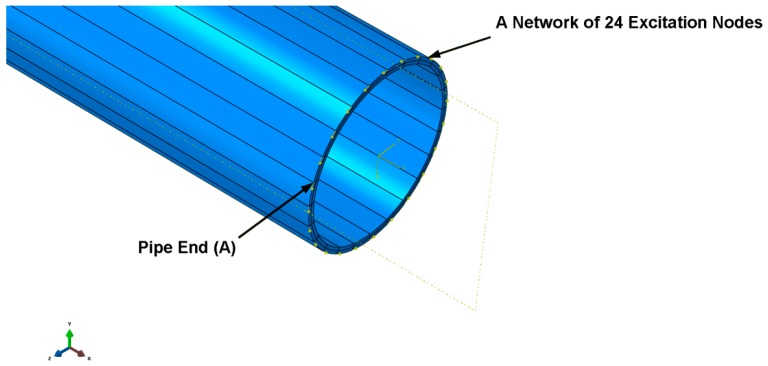
Determined excitation nodes in the simulations at the pipe end (A) for exciting guided torsional wave, T(0,1) mode, in the circumferential direction.

To simulate the GW propagation into the pipes, a particular function of excitation force can be applied to a subsection of the pipe surface at the pipe end A (the excitation nodes as shown in Figure 6) with respect to the excitation function given in (17) [88,148]:

(17) $$\begin{array}{cc} F(t)=\left\{ \begin{array}{l} 0.5(1-\cos\frac{2\pi ft}{n})\sin(2\pi ft)\\ 0 \end{array}\right. & \begin{array}{l} 0<t<\tau ;\\ t>\tau \end{array} \end{array}$$

where f represents the central frequency, $\tau =\frac{n}{f}\;$ is the signal pulse time and n signifies the pulse cycles (n = 5 was selected in the simulation). The energy of the force (load) function can be focused effectively within a finite interval in the frequency or time domain (Hilbert envelope) [88]. In the time domain, a force load can be applied to the circumferential direction of the pipe end to excite the mode T(0,1) and propagate along the axis direction with shearing motion. Mode L(0,2) can be excited in the axial direction by applying force to the axial direction of the pipe end to propagate along the axial direction with compressional motion. Therefore, both modes can be excited by applying force in different directions [149]. 

In this regard, a cylindrical coordinate system of *X*, *Y* and *Z* is required to represent the vertical, circumferential and axial directions of the cylindrical waveguide (e.g., pipe), respectively. A simple one-point node around the pipe circumference can be created as a transducer. As shown in Figure 6, the torsional mode T(0,1) can be excited using a sensor/exciter network with 24 nodes equally spaced around the circumference of the pipe surface (at pipe end A) to apply circumferential force loads. 

**Figure 7:**
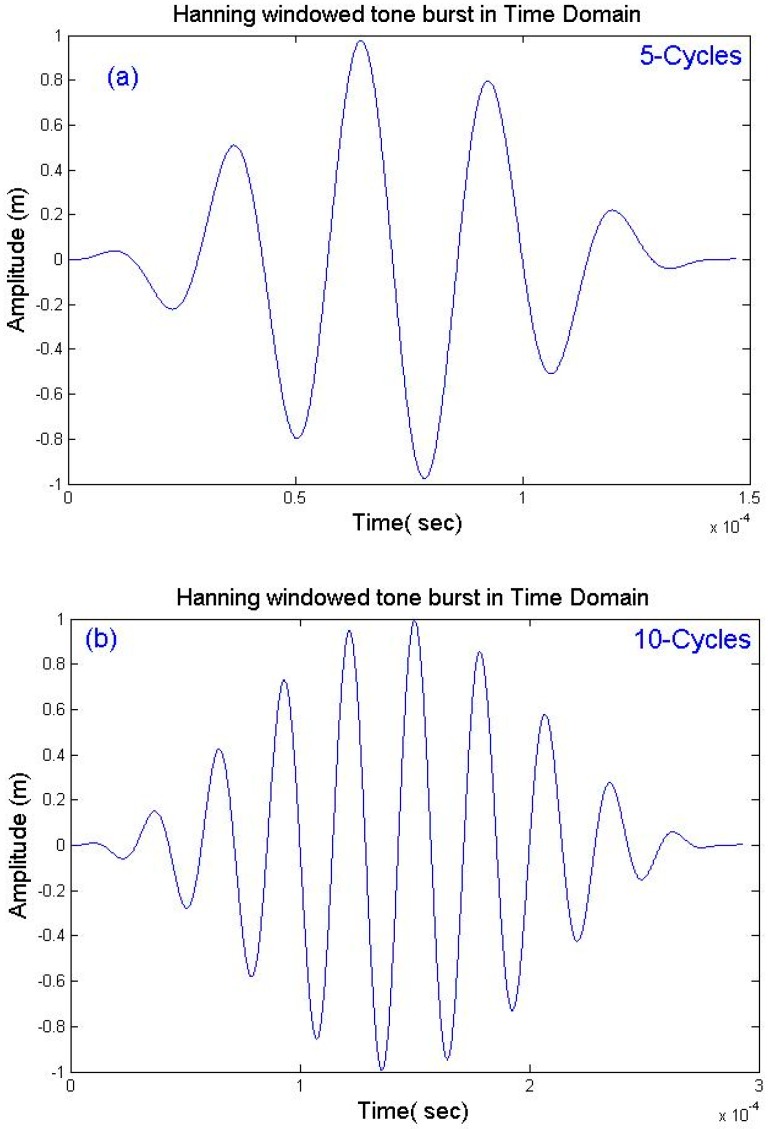
Hann-windowed tone burst at the frequency of 35 kHz: (**a**) 5 cycles and (**b**) 10 cycles.

The form of this excitation can be obtained from the force function formula (17). The graph of the signal pulse time regarding the force function is shown in Figure 7a,b. This pulse time signal was composed of a five-cycle Hann-windowed tone burst signal. Typically, narrow band signals, including a particular centre frequency, are modulated in an 8–10-cycle Gaussian window [118,150,151,152] or a 5-cycle or 10-cycle Hann-window, as shown in Figure 7a,b, respectively [2]. Thereupon, the displacements between the notch and the excitation end can be monitored. In fact, each wave can be incident on the path toward the notch, and its reflection can be detected. As mentioned in Section 4, the RC can be obtained in the frequency domain [51,56,57,128,153,154,155,156,157] or in the time domain [24,51,57,128,153,154,156,157] by dividing the signal amplitude, which is reflected from the notch, by the reference amplitude signal, which is reflected from the pipe end before introducing the notch. Moreover, the RC can be measured in the time or frequency domain by taking the maximum peak-to-peak amplitude [26].

Fourier transform, Hilbert transform and wavelet transform are three types of signal representation [158]. In using the Hilbert transform, the amplitude of a periodic signal can be extracted from the envelope of the time domain signal (waveform). Then, the signal can be employed to make the detection process easy upon the arrival of wave packets [150,159,160,161]. In the low-frequency range, the time domain is considered to filter the effects of the low-frequency response or to avoid the effects of time signal gating [24]. 

The size of the time increment must be sufficient enough to capture the shortest desired period. Hence, the fastest GW mode can be captured by an element in one step. In fact, the time increment should be at least several times less than the time of the wave propagation from one end of the element to the other end and less than the period of the input signal. Moreover, the element size should be determined accurately according to different wave velocities whilst meshing a model to ensure that one wavelet can be identified within a time step Δt. For the stabilisation and convergence of the dynamic simulations, the principal control parameter for the dynamic simulations should be selected carefully [148,162,163]. The criteria for selecting the controlling parameters of the dynamic simulation are listed in Table 2.

Therefore, five types of conditions (Table 2) should be fulfilled whilst using the explicit dynamic analysis (EDA) process to deal with the wave propagation problem.

### 4.2. Element Type, Mesh Size and Monitoring Plane

The pipe models can be discretised utilising solid elements or shell elements. The C3D8R-type node brick solid element can be used for a solid pipe model to reduce integration [164]. The application of this element reduces the total node number and output file size. 

As an example, the maximum mesh size can be determined to be less than λ/15 (as explained in Table 2; λ is the wavelength) to capture the GW propagation efficiently and obtain good accuracy. In other words, mesh size should be fewer than 15 elements per wavelength along the wave propagation direction to capture the wavelength [127]. 

**Figure 8:**
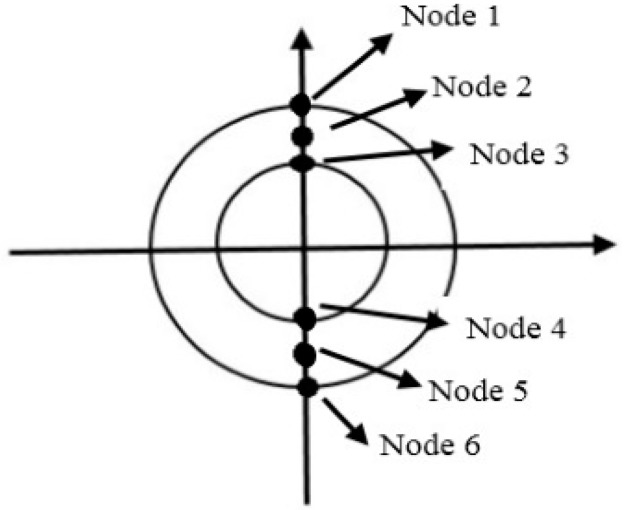
Sketch of the nodes at the monitoring plane.

A monitoring plane can be placed a distance away from pipe end A (excitation nodes) to analyse the signals reflected from defects. As an example, six nodes can be selected in the upper and lower parts of the pipes to characterise the monitoring plane (Figure 8) to investigate the excited signal and verify its reflections from defects [148].

## 5. GW Transducers

**Figure 9:**
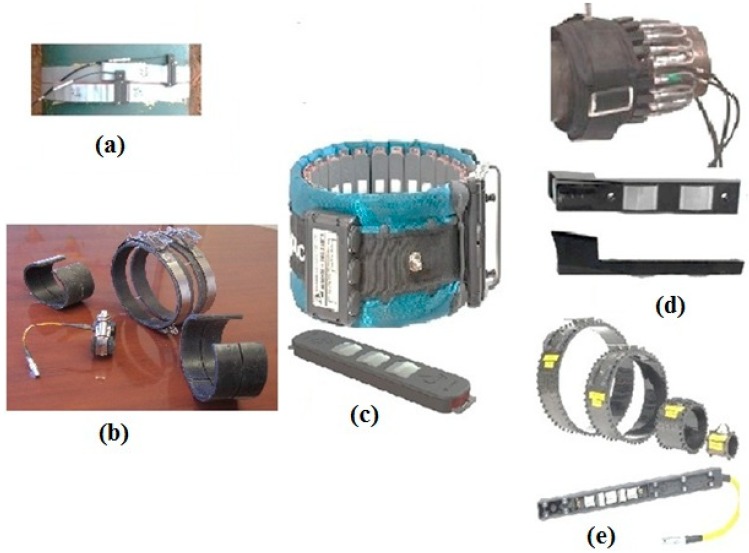
Commercially available collar and its transducers: (**a**) a US-based MsSR3030 transducer (Guided Wave Analysis LLC, San Antonio, TX, USA) [175]; (**b**) a set of magnetostrictive transducers made for high temperatures [166]; (**c**) a UK-based collar and its transducers (Guided Ultrasonics Ltd., London, UK) [176]; (**d**) a Japan-based collar and its transducers (Olympus Co., Tokyo, Japan) [177] and (**e**) a UK-based collar and its transducers (Teletest^®^ Focus, Cambridge UK) [178].

To generate and receive GWs, three types of transducers are generally used: magnetostrictive, electromagnetic–acoustic and piezoelectric. The advantage of a magnetostrictive transducer is the relatively low cost of its materials, which renders it promising as a cost-effective approach for monitoring components. Magnetostriction effects have been used for decades in various applications. Two primary effects are the Widemann or inversed Widemann effect for generating and receiving shear waves in plates or torsional waves in pipes and the Joule–Villary effect for generating and receiving longitudinal waves. The generation of shear waves that utilises the Widemann effect requires the time-varying magnetic field to be perpendicularly oriented towards the permanent bias. The Wiedemann effect is explained as the twisting of a ferromagnetic rod if an electric current is transferred along its length to create a circumferential magnetic field whilst the rod is situated simultaneously in a longitudinal magnetic field. As mentioned earlier, torsional GW modes in pipes can be generated by having one of the following fields: (a) a reversed Wiedemann effect with a time-varying circumferential field (propagation is parallel to the axial bias) and (b) the conventional Wiedemann effect with a permanent circumferential field (propagation is perpendicular to the axial bias) [165]. Waves can be directly generated in the plate provided that the plate is made of a ferromagnetic material. Otherwise, waves can be initiated in a bonded ferromagnetic strip. The considerable difference in the design of the transducer for inspecting pipes and plates is its cylindrical shape. In this case, supporting the consistent bias oriented towards the strip length by applying an external source of magnetism becomes challenging. Hence, conventional magnetostrictive sensors are designed based only on residual bias. This bias is created using a direct current coil wrapped around the magnetostrictive strip. Two ferromagnetic strips can be used in magnetostrictive transducers to provide control over the propagation direction [166]. Monitoring and conducting an inspection using magnetostrictive transducers on the basis of a magnetostrictive strip (MsS^®^) comprise two components [167]. (1) Ferromagnetic materials, such as iron–cobalt alloy or nickel, with appropriate magnetostrictive properties are used in a thin strip that can be attached to the structures being inspected. As mentioned earlier, a static bias magnetic field is required to magnetise the strip for the operation of transducers. (2) Ferromagnetic materials must also be magnetised by time-varying magnetic fields applied by a coil to excite GWs in the materials. Therefore, a magnetostrictive transducer can generate and detect time-varying strains or stresses in ferromagnetic materials. One area of application of magnetostrictive transducers is the long-range inspection of ferromagnetic tubes and pipes using GWs generated and detected with a magnetostrictive sensor (MsS). Isolated external and internal defects, such as circumferential cracks and corrosion, can be detected using the MsS technique [168]. A range of frequencies between 8 kHz and 500 kHz can be adopted to inspect the long range of pipes by using magnetostrictive transducers. For higher frequencies (up to 500 kHz), magnetostrictive transducers exhibit the potential for monitoring elbows and welds to fill in the gap between low-frequency screening and conventional ultrasonic testing. These transducers can be divided into multiple segments. The loading sequence of these segments and the number of segments involved in a signal transmission/reception can be controlled using a remote multiplexer. A transducer can be mounted 0.9 m to 1.2 m from a weld and utilised to circumferentially scan the condition of elbows/welds. A C-scan view can track an irregular condition that begins to develop in a weld. Longitudinal and torsional modes can be generated by the same magnetostrictive transducers with frequencies up to 500 kHz. Longitudinal modes render a more complex pattern of particle displacement and are more sensitive to small defects. Incoming GWs cause magnetic flux changes in the material that can be inductively detected by the coil. A commercially available GW magnetostrictive transducer (i.e., MsS, Guided Wave Analysis LLC, San Antonio, TX, USA) is shown in Figure 9a,b [166,169]. The SHM of components working at high temperatures is one the most challenging area. For this case (refer to Section 5.1), a magnetostrictive transducer (MsT) that applies the reversed Wiedemann effect can function at high stresses and temperatures caused by thermal cycling. This transducer (MsT) can also be used to test buried anchor rods, heat exchange tubes and boiler tubes [165].

Piezoelectric elements that are distributed around the pipe circumference can be used in ultrasonic transducers to generate GWs [13,48,79,170]. Commercially available transducers and transducer collars are shown in Figure 9c–e. The surface preparation of pipes is not usually needed such that in less than 1 min, transducers can be attached, and a long-distance pipe can be tested in one day [67]. The unique characteristics of GWs can be exploited using an array of transducers with special configurations. In practice, commercial devices can only monitor one flexural mode and an axisymmetric mode depending on the alignment of the transducer array. Provided that transducers are circumferentially aligned, either F(1,2) or T(0,1) can be achieved for tests using torsional modes. Meanwhile, if transducers are axially aligned, either F(1,3) or L(0,2) can be monitored for tests using longitudinal modes [95]. A circumferential array of dry-coupled piezoelectric SH transducers was proposed by Alleyne and Cawley [14]. They attached a ring of transducers as an array at the pipe end for just a single direction inspection. Notably, for bidirectional inspection, two transducer rings would be used to enable the inspector to control the wave transmission and selective reception of returned signals from the forward and backward pipe directions (i.e., commercial equipment [171]) [14]. In fact, the torsional commercial system that employs piezoelectric transducer arrays, as described previously, includes two rings of transducers arranged around the pipe [172,173]. A phase shift of π/2 should be used to excite and separate the rings by a quarter of the wavelength to cancel the wave energy in one direction and sum it in the other direction (directional control) [25,174]. In fact, the GW mode can be excited in only one axial direction. The same principle may also be used in the detection and reception of waves traveling in one direction whilst suppressing the detection of waves traveling in the other direction. Axisymmetric torsional mode T(0,1) can be excited by simultaneously firing all elements of SH transducers arranged circumferentially. Regarding this setup, the time traces at each transducer element could be recorded individually [14]. 

The interest in transducer development to enhance the testing of the structural integrity of underground pipelines has considerably increased. In meeting this objective, electromagnetic acoustic transducers (EMATs), can be employed for the excitation of low-frequency (less than 1 MHz) guided SH waves so that without an intimate contact, a source of the wave can be directly set up on the metal surface. This type of ultrasound is able to propagate and then extend over long distances circumferentially or axially (two axisymmetric families, that is, torsional and longitudinal respectively) to provide pertinent information back to the remote sensors. These transducers are applicable because of their substantial performance, decreased propagation loss and constrained waveguide space [18,77,78]. 

### 5.1. Temperature Effects on GW SHM

Damage detection in structures has been extensively studied in the NDT field. Damages have been historically detected by temporarily installing sensors on the surface of structures, performing an inspection and then removing the sensors. If further inspection is required, then this process is repeated. SHM presents an alternative to this fundamental approach. A remarkably accurate repeated measurement can be obtained if the sensors are permanently attached to the structures. This repeatability provides baseline measurement records to monitor changes that can be possibly caused by a structural damage. This approach facilitates the SHM of complex geometries and provides a high automation level. The limitation factor of this SHM approach is the difficulty in distinguishing between changes caused by damages and those resulting from environmental conditions. The major effects of changing boundary conditions, humidity and temperature can be sufficient to hide any changes caused by damages to a degree that it will probably remain undetected. Providing minimum sensor density with maximum sensitivity is ideal for any SHM system. An SHM system can be installed in accordance with the generation and reception of GWs that use piezoelectric elements as sensors. The propagation of GWs and transduction utilising PZT-5A piezoelectric material within the temperature range of 20 °C to 150 °C were analysed experimentally and numerically by Ajay R. and Carlos E.S.C. [179]. The model results showed that the time of flight of GW pulses increased with increasing temperature. However, the prediction of large enhancements in the response magnitude of the sensor exhibited a considerable gap of up to 100 °C. In addition, damage characterisation at areas located 8 cm from the actuators was not considerably influenced up to 80 °C but characterisation/detection was difficult beyond that temperature. Given that the structure’s Young’s modulus is a critical parameter, the problem beyond 80 °C can be attributed to the enhanced sensitivity of the substrate’s elastic modulus to temperature [179].

In natural gas transmission pipelines, pipe length, insulation layer thickness, task flow start and end pressures and gas composition exert considerable effects on temperature drop. In addition, temperature declines at the starting point of a long-distance gas pipeline and then increases slightly after heat transfer [180]. Ultrasonic velocity increases as temperature rises along specific directions with unique crystals’ axes. Thermal conductivity is a significant factor in ultrasonic attenuation behaviour as a function of temperature. Ultrasonic attenuation for longitudinal waves is considerably less than that for shear waves. This phenomenon indicates that ultrasonic attenuation due to phonon–phonon interaction (the major factor for attenuating ultrasonic waves in a solid at room temperature) along shear waves is the governing factor in total attenuation, which, in turn, is a governing factor in thermal energy density and conductivity [181].

To ensure the integrity of critical structures installed in difficult-to-reach locations for frequent NDT, permanently installed monitoring systems (PIMS) for GWs are potentially desirable. However, the performance of these systems has not been satisfactorily validated. Heinlein et al. [182] presented the blind trial test results of a GW PIMS system on an L-shaped 8 in. diameter pipe section with three butt welds, a radius of 1.5 D bend and an overall length of 10 m. When pipe temperature was cycled between 60 °C and ambient temperature, readings were made at 30 min intervals for a period of 40 days. Temperature variations in pipes can be a complicated factor in long-term monitoring systems. As mentioned earlier, temperature can affect GW velocity in a pipe and thus the arrival time of reflections from specific features of a pipe system includes any existing discontinuities. This condition can decrease the effect of certain monitoring techniques, such as those that use a simple baseline subtraction algorithm (BSA). The effects of environmental variations were minimised using a temperature compensation algorithm. Six simulated corrosion discontinuities were created on this pipe. The batches were used to send data to an evaluation team who did not have any knowledge of the introduction schedule or the location of discontinuities. The reports for these data were obtained before the next batch was provided. All the discontinuities were properly located without false calls, and a new independent component analysis (ICA) scheme based the SHM algorithm was applied to process the measurement data. A reduction in the effects of operational and environmental variations can be obtained using the ICA system, thereby improving system sensitivity and reducing the rate of falls calls. An increase in the collection frequency of this system is desirable [183]. The ICA system can considerably reduce operator effort in dealing with several GW measurements because it enables the extraction of features and their variations over time from an arrangement of GW measurements. Permanently installed GW monitoring systems provide a highly promising technique that can be used for monitoring critical infrastructure. Detection sensitivity was determined to be approximately five factors better than that commonly provided in one-off GW tests. Discontinuities before and after the 1.5 D bend were detected at a similar stage [182].

Vinogradov et al. [184] used a magnetostrictive probe for a mock-up pipe at 200 °C. High amplitude was constantly obtained over a period of 270 days. An average SNR within the range of 44–47 dB enabled the detection of 1.5% defects in an open span area of the pipe before BSA was applied. The use of BSA allowed the suppression of a huge amount of signals that involved pre-existing conditions at 23 dB. A random fluctuation of the coherent noise level of 4% CSA was obtained to specify a practical limit for method sensitivity at 0.8% CSA. In summary, temperature effects were determined as follows. (1) The reflection from the MsT transducer was relatively high at low temperatures and completely disappeared at high temperatures. (2) No related variation in the reflection amplitudes from known defects (1.5% and 2.5% defects) were observed at constant temperature regimes. (3) No considerable variation due to temperature was found in the relative amplitude of either random or coherent noises. (4) The dead zone from each side of the MsT increased from 0.3 m at 20.5 °C to 0.45 m at 202 °C at 90 kHz. (5) The total loss of amplitude received from a weld signal due to elevated temperatures was calculated at 9 dB. This effect is mostly relative to a decrease in energy transfer at an increased temperature due to epoxy softening. (6) Attenuation increased from 0.492 dB/m to 1.92 dB/m at 64 kHz. (7) GW velocity dropped from 3175 m/s at 20.5 °C to 3063 m/s at 202 °C [184].

## 6. Common GW Testing Methods

Corrosion is the most common cause of pipe failure. Pipes carrying hazardous or corrosive chemical content must be inspected at particular periods to identify any corrosion early. In this way, systems can be shut down, and the remaining thickness of the pipe wall can be measured before any catastrophic failures or any potential leakage occurs. As mentioned previously, metal loss features, such as general corrosion, decrease the inspection range of GWUT because of the scattered wave energy caused by rough corrosion surfaces that attenuate the propagating waves. By contrast, defect size can decrease the inspection range that is to be identified. Here, commercial GWUT techniques can be employed to monitor erosion, in-service damages, and any external or internal corrosion occurring on the pipe wall because it requires less preliminary work (i.e., removing insulation or performing excavation) than other NDT techniques. 

The commercially available instruments use the pulse-echo system and are designed to send axisymmetric modes either the longitudinal or torsional wave (e.g., L(0,2) or T(0,1)) using an array of transducers or magnetostrictive sensors attached to a pipe surface [95,178]. 

According to standard BS 9690-1:2011 [93], GWT instruments use multiple rows of transducer elements to achieve mode selectivity. In this regard, inspection using the L(0,2) and T(0,1) GW modes require four rows and two rows of transducer elements in practice, respectively. For L(0,2), the direction of motion should be selected, and the L(0,1) mode should be rejected by adding additional rows. Hence, two transducer rows are sufficient to select between L(0,2) and L(0,1) modes, and two transducer rows are adequate to select between two different directions of motion. However, for T(0,1), mode rejection, so the two rows are sufficient only for motion–direction selectivity [93]. The axisymmetric modes (L(0,2) or T(0,1)) propagate along the pipe length and interact with the defects and pipe features, resulting in the reflection of some of the incident wave energies to enable inspectors to detect defects. 

As mentioned previously, the incident wave energies may be mode converted into non-axisymmetric flexural modes. Then, the converted mode and directly reflected signals are received together at GWUT systems to record and process the received signals following the measurement of the axial position of a defect. To address any defect (i.e., corrosion) or severity issue using GWUT, Plant Integrity Ltd. (Cambridge, UK) put forward a classification graph, in which a score is given to responses in accordance with their circumferential expansion from the focused test and their axisymmetric test amplitude [95,178]. Distinguishing axisymmetric features, such as flanges or welds, from localised metal loss features, such as corrosion, is discussed in Section 3. Current commercial devices can only monitor one flexural mode and axisymmetric mode regulated by the alignment of the transducer array (axially aligned transducers for longitudinal tests or circumferentially aligned transducers for torsional tests). As already mentioned, current systems can distinguish horizontally and vertically flexural modes from axisymmetric modes by comparing the received signals from the segments of the transducer array and the feature types can be drawn as conclusions. In this regard, the amplitude of each time-dependent series can be assessed and the CSA of a defect can be estimated from the amplitude of axisymmetric signals, and the plane (i.e., vertical or horizontal plane) of the defects related to the orientation of transducer arrays can be predicted [95].

The improved test methodology is summarised in three steps: (1) performing unfocused long-range ultrasonic testing (LRUT) applying axisymmetric wave modes and using improved methods for showing the results to detect regions of interest, (2) performing an inspection using the phased array flexural mode focusing technique at axial locations specified by the data obtained using Step 1 and (3) combining the angular distribution data obtained using Step 2 and the amplitude data from the normal inspection technique to provide a classification of defect severity [95]. These steps are discussed as follows. 

First, a ‘defect category’ denoted as *C* is determined for the axisymmetric amplitude from the focused test response. In this regard, distance amplitude calibration (DAC) lines are drawn on the basis of the received signal amplitudes from welds to define three different categories for the identified defects: all amplitudes that are less than −12 dB from a weld signal are categorised as *C* = 1, the amplitudes between −6 and −12 dB are categorised as *C* = 2, and the amplitudes greater than −6 dB are categorised as *C* = 3. Indeed, a relationship exists between the defect size and the amplitude of the received defect signals such that large reflection responses occur when the defects are likely to be large [185]. The defect shape and its orientation may affect the amplitude of reflection responses. However, the reverse is not necessarily true [112]. A worthy consideration is that on the one hand, a maximum signal received from a defect will result when the transducer angular position matches the defect location. On the other hand, minimum signals will result when the transducer angular position is out of line with the defects. The result achieved using the Teletest screening equipment shows that the technique can be effective at the circumferential position of small defects on the pipe being investigated. Hence, it can enable an operator to decide to conduct a focused test at a specific location [95]. The tests performed by commercially available equipment require a transducer with only eight 45° segment arrays. The drawback is that variations in the phase velocity of different wave modes are not accounted for. This feature limits the accuracy of the results. However, the same is not true empirically. This difference may be due to wave modes with various phase velocities that are likely to be of higher order (n > 5) and travel at much lower amplitudes so that they can be considered negligible in the overall results [95].

Second, additional information can be provided to examine how localised the responses are in relation to the pipe circumference. This provision may be achieved by running focused tests so that the results are plotted on a radial polar response plot. In fact, by focusing the energy of ultrasonic waves at a desired angular position and distance along the pipe from the transducers, the angular distribution can be calculated. The ‘directionality distribution’ is calculated from the focused test and described graphically. The defect response is recorded at each location around the pipe in 45° steps of incremental focus. On the basis of a given response amplitude on a narrow part of the pipe circumference, the defect is highly localised so that it is probably a severe and deep defect [112]. 

In terms of the angular distribution resulting from the focused response, a classification of directionality distributions is defined between 0 and 3 in accordance with how localised the responses are. The defect is classified as directionality (D) = 3 in which its angular distribution is less than 45°. In other cases of focused response, defects can be classified as directionality (D) = 2 with angular distribution between 45° and 90° or directionality (D) = 1 with angular distribution between 90° and 315°. Directionality (D) = 0 can be defined for a girth weld with the angular distribution of 360° [112,185]. 

Finally, a defect classification ‘follow-up’ (F) priority operates as the product of D and C, as shown in Table 3 [112,185].

In a different case, the Wavemaker WavePro software was used to generate a report for a 4 inch pipe which was painted in epoxy and sited next to a road crossing. In this case, an inspection range of around 20 m was achieved on either side of the transducer location. The DAC curves for the welds were computed on the basis of the welds identified by the software. Thereafter, the defect call level was calculated by comparing the calculated output amplitude and the weld echo level. The calculation yielded a −14 dB reflector (this value was considered in accordance with the normal size of a weld cap) as an average site weld concerning the amplitude of the incident wave. A red component represented a non-axisymmetric mode-converted signal, whereas a black component represented the received axisymmetric signals (Figure 4). The case in which the detection result showed red and black signals but with the red signal (mode-converted signal) being greater than the black signal (reflected incident mode) revealed the possibility of corrosion at the entry location of the road crossing [22,53,67].

Some other cases may cause low attenuation (no attenuating coating or heavy general corrosion), and the density of the piping features, such as bends, tees and infrequent welds, may also be low. This case shows that multiple signals received from separated welds properly record a low level of noise in each direction from the location of the transducers. In addition, the case reported here illustrates a clear received reflection from a problem located away from the weld indications. Hence, the interpretation of received signals is relatively simple and could be reported by experienced NDT specialists who had completed a one-week training course with an additional week of field testing under supervision [53,67]. However, a problem occurred with the set of DAC curves. One of these curves was a pipe road crossing with bitumen coatings or embedded in concrete. These features resulted in limitations in the evaluation of the reflections arising from the buried area. This case required a highly skilled and experienced technician.

In some instances, the aforementioned difficulties may be addressed by testing the same pipe from the other side of the road [22,53]. In accordance with the ASTM E2775-11 standard [186], a qualified and certified personnel is required for GWT as determined in the purchase order or contract. Qualifications shall consist of specific training regarding the use of equipment, interpretation of test results and application of GW technology [186]. In other cases, a report is generated using the *MsSR3030* software. A 128 kHz torsional GW mode was used by applying MsS to inspect a welded pipe support in the insulation. A strong interaction occurred between the welded pipe support and the low-frequency GWs. Conducting an inspection at less than 60 kHz resulted in a false call which was caused by a large trailing signal following the signal of the pipe support (at 32 kHz torsional data). In one case, the wavelength of a 128 kHz torsional mode is four times shorter than that of a 32 kHz torsional mode. Under such condition, the pipe supports and the 128 kHz torsional mode do not interact. Thus, 128 kHz signals would be effective in detecting any defects in the area following pipe supports. This finding indicates the necessity of inspecting pipelines with welded pipe supports using high frequencies [175,187]. 

Detecting, locating and sizing defects by applying ultrasonic GWs represent a wide subject area and involve intensive critical subjects in the non-destructive testing research field [47]. For defect detection and corrosion monitoring by using GWs in pipes, information is available in [5,13,24,28,40,52,53,74,105,111,121,188,189]. The results of the numerical simulations and experimental studies of the aforementioned research have been published. Many of such studies have been particularly adapted to satisfy the requirements of identifying defects, such as cracks, and their characterisation in pipes [8,14,38,56,57,68,190]. For instance, Kim et al. [60] and Fletcher et al. [58] proposed an approach to account for the identification of axial cracks by using horizontally polarised SH waves and focused guided ultrasonic waves, respectively. Moreover, the characterisation of circumferential cracks was addressed in the works of Ditri et al. [191] and Mu et al. [71,192]. Pipeline defect detection, sizing and characterisation by GWs are noted in the references [71,72,79,80,193]. To locate cracks and perform sizing in a hollow isotropic cylindrical structure, Valle et al. [57] used circumferential WGs. In this context, the length of a crack (or multiple cracks), including the frequency spectrum of the RC, was calculated by using an improved Auld’s formula. Then, by adopting a time-frequency DSP operation technique (time domain analysis) on the backscattered signals and comparing the new and previous results, the crack could be located. Crack locating and sizing using this technique were dependent on the input signal frequency. However, as mentioned previously, the estimation of the remaining pipe wall thickness is not currently possible. In recent years, the GW phased array method has been examined to improve the angular (circumferential) resolution of tests.

### 6.1. Higher-Order Modes

As mentioned in Section 2.3, the second integer of the modes shows a counter-variable or family of modes. This integer roughly shows the mode of vibration within the cylinder wall. In this issue, the basic modes that can spread at zero frequency are assigned the value of 1, and the higher-order modes (i.e., flexural modes [194]) are given consecutive numbers. As an example, mode F(1,3) represents the third flexural mode of the circumferential order one [91]. Higher-order modes require a minimum frequency thickness. The first frequency is known as the ‘cut-off frequency’ of a wave mode, and the specimen does not provide support below that mode. In addition, higher-order modes provide a further complicated displacement pattern relative to basic wave modes. Hence, in a number of applications, they are not evaluated or are even intentionally suppressed due to this complexity, but a particularly helpful information can be expressed through an analysis of [112]. However, in 2009, Cotton [112] demonstrated that higher-order modes are actually dispersive, resulting in a decrease in their amplitudes with propagation distance. This condition spreads out the pulse over time, thereby leading to a low amplitude. Hence, filtering a specific arbitrary mode from multimodal signals to be used for defect detection is interesting. A similar research was conducted by Balasubramaniam et al. [9]. Corrosion detection and sizing for defects on pipe specimens were investigated by using dispersive and non-dispersive higher-order mode cluster (HOMC) of guided ultrasonic wave propagation in the pipe circumference (circumferential direction). In this regard, the identification of effective modes for each type of corrosion (i.e., pitting corrosion and axial stress corrosion cracking) in the region of the pipe support locations, as well as their defect characterisation sensitivity, was investigated. The HOMC of GWs can be successfully applied to locate, image and size artificially machined defects (i.e., pitting corrosion).

In 2007, the GW HOMC in the high-frequency range was used by Satyarnarayan et al. [8] to investigate the problems concerning pinhole and crack detection and sizing in pipe support areas [47]. They showed that when a non-dispersive GW HOMC interacts with defects during propagation along the circumferential direction in the pipe support regions of a mild steel pipe specimen, a small dispersion was received at the relatively high frequencies. The higher-order modes were excited by using a 2.25 MHz linear phased array transducer and a 1 MHz conventional circular transducer. These transducers were normalised to the reflection signals received from the largest defects. In this study, the expected amplitude of the theoretical signals was compared with the amplitudes received experimentally from particular notches. The percentage error was also calculated. The findings revealed that the dispersion significantly reduced at 2.25 MHz. Less dispersion in the received signals at 2.25 MHz was observed using conventional and phased array transducers in comparison with the dispersion observed in the received signals using a conventional 1 MHz transducer. In addition, less noise contamination (high SNR) was noted in the signals received by the phased array transducer than in the signals received by a conventional 2.25 MHz transducer. This result benefitted the presentation of an improved imaging for small discontinuities through the pipe CS so that the size of the defects was successfully calculated by using the amplitudes of the received signals. The results showed that detecting, locating or even sizing simulated axial corrosion crack-like defects, as well as pinhole-like (pitting) corrosion defects, are possible. 

On the basis of the HOMC approach, Shivaraj et al. [13] investigated the monitoring of hidden pitting corrosion at inaccessible pipe supports without lifting or disturbing the pipeline layout arrangement. They excited the GW HOMC by applying piezoelectric transducer arrays. The same results were found in their survey for pitting-type corrosion (small defects of 1.5 mm in diameter) detection by applying circumferential GW modes. The mode selection criteria for the dispersion curves at high frequencies were investigated, and the defect CSA and defect characterisation were studied using an energy plot [13]. However, implementing an inspection for the detection of small defects in the high-frequency ranges was difficult due to the presence of multi-mode dispersive waveforms. 

In 2016, Khalili and Cawley [195] showed the potential of a single lamb wave mode excitation with low dispersion at a frequency thickness of almost 20 MHz-mm. Through FE analysis and experiments, they revealed that the HOMCs are most probably simple signals so that a signal dominated by the A1 mode can be generated even in an area where numerous wave modes are present with similar phase velocities. In fact, the A1 mode excitation was imposed due to its non-dispersive nature and minimum surface motion at high-frequency thickness products that tended to be rather unaffected by attenuative coatings and surface roughness [195]. These characteristics were close to those of the HOMC described by other researchers. A similar configuration was used by Balasubramaniam et al. [8,196,197,198,199,200] to achieve what they expressed as an HOMC. In their studies [196], the HOMC deflected shape was considerably close to the shape of the A1 mode, and the A1 mode group velocity was within 2% of the group velocity of the reported HOMC [195,196]. 

### 6.2. GW Focusing Technique

Unfortunately, small defects cannot provide responses with a sufficiently large amplitude. At the same time, large defects do not necessarily have responses with large amplitudes. Thus, these limitations cause the GW methodology to be unreliable if GWUT only uses the amplitude for quantitative inspection. Therefore, constructing an improved spatial resolution by using a complementary technique is required so that detailed data about the angular position and size of defects can be obtained [95]. 

A GW ultrasonic focusing technique has been studied and adopted for anomalies (defects such as cracks or corrosion) [14,58] to be detected, located and sized in industrial pipelines. The advantages of this technique include inspection over long distances, high detection sensitivity to structural anomalies, provision of information about the extent of the defects and their circumferential position [95], provision of information about the resolution of multiple defects and the potential for further detailed assessment. In addition, this method can raise the impinging energy, enhance the inspection resolution, increase the propagation distance and locate defects [35,71]. This technique is also an economical and useful NDE method for the inspection of pipeline defects [201]. Some of the focusing techniques have been examined by several researchers over the past 10 years, e.g., (1) time reversal focusing method (TRM), (2) analytical dispersion focusing (AD focus), (3) natural focusing (non-axisymmetric flexural mode focusing), (4) phased array focusing (5), angular profile tuning (APT) focusing (6), Signal-based focusing (SBF) and (7) synthetic focusing [14,15,35,58,71,80,81,131,202,203,204,205]. 

When a defect signal is identified in a pipe, it can be reversed in the time domain. Then, the time-reversed signals can be retransmitted to concentrate (focus) the wave energy locally at the position of a defect. This method is called the TRM [128,206,207,208]. Previous investigations have revealed that this technique overcomes the problems that arise due to dispersion and that it can be useful in highly scattering environments [207]. However, this method needs a valid preconception of the defect location, and it cannot be applied to build a focus at an arbitrary location [95]. 

As a solution to the abovementioned disadvantage of the TRM, an analytical alternative method called AD focus was developed by Sanderson et al. [209]; this method enables the mathematical simulation of the received signals from defects at an arbitrary angle or axial location on an isotropic pipe. Then, the simulated signals can be time-reversed and retransmitted to generate a focus at any location on the pipe, along with the consideration of dispersion [112]. Nevertheless, the method suffers from an inadequately controlled level over the ultrasound and test directionality as propagation exists from the transducers in both directions, thereby producing misleading and confusing results [95].

A partial loading can be applied around the pipe to generate non-axisymmetric flexural modes. This loading naturally focuses ultrasonic energy at certain distances. This type of focusing is called natural focusing [128,210]. In other words, the focal spots of natural focusing are points in a pipe where the intense constructive interference of several waveforms occurs due to partial loading around the pipe circumference or, in numerous cases, the crossing of the elbow area by a number of waves [51]. However, some defects may be lost at some positions because the distribution of ultrasonic wave energy is not enough at that point to be focused. In fact, the degree of partial loading surrounding the pipe circumference, focal distance and frequency can change the natural focal points [210]. Hence, in practice, if an inspector were to only use the focusing technique, then the results achieved would be erroneous. As an example, the angular profile of the excited mode T(n,1) demonstrated for a schedule 40 steel pipe (8 inches) and a source loading of 90° circumferential length at 60 kHz is focused at the top of the pipe at an axial distance z = 702 inches. However, most energy is distributed at the bottom (z = 342 inches), and some defects may not be detected at z = 702 inches [210]. Furthermore, the frequency can change the shape of the angular profile as well as the natural focal points [207].

In 2001, Rose et al. [211] published a paper in which they described that a high control over the GW modes can be gained if a phased array transducer surrounding a pipe is used instead of natural focusing. In fact, the focusing transducer tool is divided into segments around the pipe circumference so that mode tuning implementation or focusing can be achieved by applying appropriate time delays to each segment. This method is similar to the technique that generates a focus from a high-frequency phased array transducer, and the focusing tool is regarded as a phased array whereby the exact position of the constructive interference can be selected and a local concentration of the energy may be achieved at an arbitrary circumferential or axial focal point [15,51,95]. Therefore, the system can be programmed for high-speed multimode industrial inspection, and the modes can be adjusted by digital inputs [211]. All the collected data from each segment are then combined to create a polar plot of the received amplitudes. Hence, this type of focusing can provide additional information to examine how localised the responses are in relation to the pipe circumference [15,71,95].

The phased array focusing technique can focus ultrasonic energy at a location on the pipe circumference where the defect is located to enhance the responses from it. In another case, the implementation of the focusing technique based on non-axisymmetric flexural modes in a pipe in accordance with the NME method was developed by Li et al. [212]. They indicated that the energy distribution of non-axisymmetric flexural modes for circumferential distribution is non-uniform around the pipe. This non-uniformity of the distribution represents an opportunity to tune the angular profiles of the generated GWs through the arrays of transducers to possibly increase the detectability of pipe defects. As the superposition of all of the excited GW modes with different phase velocities covers the whole pipe, the angular profile along the propagating direction can be changed if a small change occurs in the mode phase velocities. Thus, phase-matching changes between different modes can possibly affect angular profiles [51]. Furthermore, the predicted angular profile can provide additional information about the determination of the ideal transducer location in a pipe to detect defects in a particular position [15,47,128,164,204].

Mu et al. [71,80] studied circumferential extents, the circumferential location and the depth of different defect shapes. For instance, a volumetric elliptical corrosion, a planar saw cut and a volumetric through-wall hole distributed at distinct circumferential angles and axial distances from phased array transducers can be detected by applying the GW phased array focusing inspection technique. They considered 44 circumferential focusing (focal) positions (44 elements, e.g., three rings of transducers divided into four segments around the pipe and then at a particular axial distance relative to each circumferential defect around the pipe circumference). Focusing at 44 angles was accomplished so that a record of the defect echo of the maximum amplitude was determined. In other words, through the use of the NME computational technique, APT can be acquired by measuring the focused energy of the GWs impinging onto circumferential defects, including different CSAs [71]. Therefore, the reflected energy regarding each focal angle can be plotted so that a theoretical reflection profile of reflected signals is generated [71].

According to the other works of Mu et al. [80], the reflections from differently shaped defects but with a similar CSA are different. According to Li et al. [213], the APT focusing technique can be implemented experimentally to focus the non-axisymmetric GWs which are generated using multichannel transducers. This case can result in the detection of small defects. Moreover, effectively measuring the defect circumferential extent is possible by comparing experimental and theoretical profiles [71]. However, the APT-based focusing technique only works under unfeasible conditions because the pressure distribution of transducers can be imagined to be uniform. An angle beam transducer which is attached to a slide wedge and accurately mounted on the pipe surface cannot be described properly by the theoretical prediction of an acoustic field on the basis of the NME method [212,214]. Moreover, the abovementioned technique requires an adequate number of transducers to enhance efficiency related to detectability and to control the modes in the selected range of frequencies [15].

Despite these drawbacks, Mu et al. [215] showed the scanning results of a non-axisymmetric GW constructed pipe image. Hence, the detection of multiple defects by using this technique can be achieved through the interaction of GWs with defects that change the boundary conditions and consequently lead to wave scattering which can be received by transducers [35,71,81]. To enhance the defect determination at a circumferential position of the pipe from the defect echo, Zhang [128] considered a total of eight focal spots in a 35 kHz T(n,1) group. He focused on eight different circumferential angles that were moved at a specific distance from a saw-cut notch at 45° with 3.6% CSA in a 16° schedule 30 steel pipe to detect and locate defects more accurately than by applying only four focal spots. In focusing at four (0°, 90°, 180° and 270°) and eight (0°, 45°, 90°, 135°, 180°, 225° and 270°) points, different circumferential angles indicate that the circumferential position cannot be determined accurately from the minor defect echoes by using only four focal spots. In fact, when focusing at 0° and 90°, weak reflection echoes from the saw-cut defect with circumferential extent at around 30° were received. By contrast, when eight focal spots were used, the approximate defect location with a maximum amplitude at 45° was obtained. In accordance with the GW generation system with multiple channels, the excitation condition provided on each channel is the same, and the energy distribution (focused angular) profile on each channel can be expressed mathematically as follows [35]:

(18) G = A ⊗ H

G expresses the focal energy profile function. One single excitation element can generate a discrete angular profile, which is denoted by H. For the excitation channel, the complex symbol A represents the discrete weight function. 

If G is 1 at the focal spot in the case of focusing, then the calculation of A is expressed as follows:

(19) $$A\;=\;1\;{⊗}^{-1}H\;=\;{\mathrm{FFT}}^{-1}\;(1/H)$$

where FFT^−1^ and ${⊗}^{-1}$ express the inverse fast Fourier transform and deconvolution operator, respectively. The amplitude factor c_i_ and the input phase delay θ_i_ regarding the i-th excitation channel (transducer) denote the amplitude and phase of A_i_ (the corresponding weight function), respectively. Therefore, time delays concerning each channel input can be calculated as:

∆t_i_ = −θ_i_/2πf (20)

In 2011, Kang et al. [15] introduced another approach to increase the possibility of defect identification in pipes by using the SBF technique. With this technique, GW can be generated using transducer arrays. This method is similar to the APT focusing technique, but it achieves excellent performance experimentally with regard to the dispersive nature and the effect of the material through cross-correlation analysis. In addition, the SBF method is highly advantageous in terms of the limited hardware conditions. In this case, an ultrasonic GW can be focused when a sufficient number of transducers are available to provide a perfect mode control. However, the SBF technique is only application in pre-emitted pressure fields. That is, the SBF technique is not applicable when a beam is focused on a region of a pipe without any defects. To discover additional details about the SBF technique, the reader is referred to the investigation of Kang et al. [15]. 

Zhang [128] employed 3D explicit dynamic FEM to investigate the potential of GW focusing for small defects such as a planar saw cut and a spherical indent in hollow cylinders. The Abaqus commercial software was used to evaluate the shape of the elements and to apply a primary mesh. In addition, at least two or three displacement elements with a dimension of roughly 10% of the wavelength were applied to ensure the convergence of the FEA and the GW torsional mode group, T(m,1), at 100 kHz, which was focused successfully beyond a number of minor defects in a schedule 40 steel 4 inch pipe. The focal spots in the schedule 40 steel 4 inch pipe were at an axial distance z = 48 inches. In addition, natural focusing was applied in angle tuning and frequency. The natural focusing technique was also used to investigate a hollow cylinder by implementing a partly loaded circumferential excitation. With this technique, the energy of the guided ultrasonic wave could be focused naturally at several distances on the top or bottom of an 8 inch schedule 40 pipe at the axial distances of z = 702 inches and z = 342 inches through the excitation of torsional group modes T(m,1) at 60 kHz by using a transducer with a 90° circumferential length. However, the defects in these locations could not be detected properly. Nevertheless, at the focal spots, the ultrasonic energy could be increased, particularly if the natural focal spots could be relocated through the structure. Therefore, natural focusing was found to be an improved technique to enhance the results of GWs for pipe inspection [128]. 

On the basis of the work of Davis and Cawley in 2009 [14], the focusing technique was previously selected for the acquisition and delay laws which were used in each array of transducers such that the focused energy could be physically acquired at the preferred focal points. This technique not only takes time, especially when the whole pipe wall is required to be covered, but also requires large hardware due to the need to separate amplification and signal generation on each transducer array. Furthermore, a synthetic focusing post-acquisition technique was investigated by Davis and Cawley [14]. In this approach, they utilised piezoelectric transducer elements to form circumferential arrays so that the arrays excited torsional GWs and interacted with pipe features as a reflector. This process included defects or weld caps; the reflecting features being imaged by the recorded, backscattered, synthetically focused modes were applied to every desired position in the pipe wall through a backpropagation algorithm. Therefore, in estimating the depth of the defects, the image amplitude of the defects was used as a way to approximate the circumferential extent. The entire width at half maximum of the circumferential profile of the defect image was utilised. By contrast, by using an imaging technique, defect information can be directly obtained from reconstructed images. However, similar to the GW inspection technique that uses axisymmetric modes, the synthetic focusing technique provides a lower SNR than the real-time phased array focusing technique [14,47]. 

## 7. Limitations and Capabilities of GWUT

A standard pipe has several features, including welds, pipe supports, pipe branches, long straights and bend sections, all of which may be affected by some type of metal loss feature. The GW technique has been developed as a fast screening tool to monitor the features of metal loss, such as erosion, corrosion and in-service damage, by covering 100% of the pipe wall [128]. Therefore, this technique is suitable for inspectors as it will save them time in carrying out a detailed inspection of suspect areas. As mentioned in the Introduction, the GWUT technique is an applicable inspection method for inaccessible pipes, such as those that are clamped, sleeved or buried. This method also covers cased pipes where scaffolding or rope access would be required for inspection using conventional NDT, bridge piers, under supports, road crossing sections of pipelines, painted pipes, spirally welded pipes, offshore risers, high-temperature pipelines (< +125 °C), stainless steel pipes and corrosion under insulation in the oil, gas and petrochemical industries [2,17,18,19,20,21,22,23,24,26,27,28,170]. The GWUT technique can also be employed in dealing with offshore platform jacket structures, railway lines, cables and wind turbine towers [29,30]. Conventional NDT inspection methods are used to assess the details of highlighted metal loss in suspected areas in a pipe, which has been identified using the GW technique. The result potentially provides a qualitative measurement of any wall loss defect. For example, the visual inspection method can be applied to detect external defects, and ultrasonic thickness gauging can be used to detect internal corrosion. In fact, wall thickness measurement cannot be performed directly by using GWUT. Nevertheless, GWs are sensitive to the depth, axial and circumferential extent of metal loss defects due to the circular wave propagation along the pipe wall and the wave interaction with the annular CS [22,41,42,43,44,45,46,47,53,95,216]. The range of frequencies below 200 kHz renders GWUT suitable for inspecting long-distance straight pipes. The inspection range typically observed lies between 20 and 30 m in both directions from the transducer location. With regard to the straight welded pipes with straight joints, the test range has increased to a 300 m coverage from the transducer location (150 m in each direction or even more in specific cases) so that an inspection range of 1 km or more per day is not so far-fetched [53,217]. Table 4 shows that the inspection range may be decreased as the overall pipe conditions deteriorate. This case is discussed in the next subsection [178]. 

### 7.1. Attenuation and Effects of Pipe Features, Pipe Surface Condition and Surrounding Materials

General corrosion (rough surfaces) can cause energy scattering and decrease the inspection range. Generally, the inspection range of GWs propagating along a pipe is a function of the pipe attenuation rate and pipe features encountered. As an example, defect identification is not reliable after some welds in each direction from the transducer location (even if signals from further welds are received) due to the decrease of SNR. Considering that the propagation of GWs is not possible after pipe flanges is necessary [21,45,53,178,217]. As mentioned previously, a common GWUT application is the inspection of straight pipeline sections in a forward or backward direction. However, the GW distortion caused by testing around elbows or sharp bends in both directions can reduce the capabilities of the tests [11,22]. In most cases, no difficulty is encountered when inspecting pipes after passing any pulled or swept bends. Moreover, branch lines should be inspected separately from the main pipeline [22]. For the long-length inspection of pipes with external coatings, such as mineral wool pipe insulation, no difficulties are presented with the use of GW technique, and the inspection range is reduced when foam polyurethane insulation causes ultrasound losses [22]. A limitation of the GWT is observed for pipes encased in cement and passing through concrete walls. Concrete may affect an operation by rapidly attenuating the ultrasonic wave energy. In addition, viscoelastic coatings such as Bitumastic 50 can highly attenuate waves [218]. Some heavy adherent wrappings can attenuate ultrasonic wave energy and may have an effect on the inspection operation (Table 4) [22,178]. 

In the case of pipes with extreme viscosity content (e.g., heavy scale deposits inside the pipe), GWUT would not be effective as an inspection method. In other situations, such as sub-sea inspection, GW equipment is also not applicable. Nevertheless, ultrasonic waves can travel through long immersed pipes on the sea bed. These difficulties are results of large increases in signal attenuation in a pipe due to numerous factors:

(1) Leakage, such as bulk wave radiation, into the surrounding material such as soil (the attenuation is not often severe) or in concrete provided that it is strictly attached to the pipe.
(2) Mode conversions, such as branches or bends, cause the mode conversion or reflections of the forward or backward propagating symmetric wave modes into non-axisymmetric modes.
(3) Reflection caused by features such as welds decreases the traveling signals.
(4) Material attenuation, e.g., bitumen as a high lossy coating.

Attenuation due to rough surfaces and lossy coatings can be decreased by inspecting at low frequencies (25–70 kHz). The test range can often be extended provided that low-frequency transducers are applied. However, using these transducers affects defect sensitivity and spatial resolution [22,53]. Test frequencies could be optimised to inspect pipes with lossy coatings. In this case, the propagating wave has small energy in the coating. Moreover, testing buried pipes in concrete is possible at high frequencies in which the wave energy is carried in the internal surface of the pipe. In inspecting buried pipelines, an intelligent pigging method is another possible alternative to GWT. However, this process may cause production disruption, and it involves high costs, especially in excavation sites that would lead to disruptions of passage (e.g., rail or road crossings). Nevertheless, GWT enables the buried pipeline inspection of an uncoated section especially for testing rail or road crossings. In such cases, the pipe needs to be inspected from both sides of the crossing. Buried pipe inspection is limited by the following:

(1) Presence of surrounding materials (e.g., thick bitumen coatings)
(2) Contact with soil
(3) Presence of complex geometries such that each geometry leads to GW attenuation

Determining the impacts of soil, sand, coatings (e.g., thick bitumen coating) and concrete as materials surrounding buried pipes is important for defect detection and characterisation, for which selecting proper modes and predicting power penetration are crucial. 

SNR has a key role in pipe damage detection capabilities [31]. Deducing the anticipated GWT test range from attenuation values is possible because test ranges enable the reflected signals from defects to travel back to the ring of transducers. Therefore, the essential physical parameters controlling ultrasound leakage from buried pipes need to be accounted for in the analysis. This condition can provide a basis for the design of new coatings of buried pipes with the aim of increasing the test range. Only experienced operators should be appointed to test buried pipes [219]. 

The discussion concerning embedded structures is separated into the following:

(1) Strongly loaded waveguides
(2) Weakly loaded waveguides

The first group includes structures such as bars or pipes embedded in rock or concrete as the surrounding materials, which significantly cause the attenuation of GWs and influence the properties of GWs. Thus, the acoustic impedance of the waveguide materials has a similar magnitude level to that of the surrounding materials [22]. In this case, the wave mode shapes and the velocity dispersion curves can be changed so that new modes may appear or be lost. 

The second group includes structures such as steel pipes underwater. As the acoustic impedance of water is considerably lower than the acoustic impedance of steel, the energy of GW modes leaks into the water, but the mode shapes and the velocity dispersion curves are almost unaffected. In practice, road crossings and buried pipes are considered in this classification. Changes in the surrounding materials resulting in wave scattering, in addition to changes in the dispersion curve or wave attenuation, should be considered [21,22,53]. 

### 7.2. Guided Waves for Defect Detection in Coated Buried Pipes

Soil and coating affect GW propagation inside pipes. These effects must be understood for the proper selection of modes and prediction of penetration power for characterisation and defect detection. In addition to successfully using GW techniques for aboveground pipe inspection, attempts to extend the technique are being initiated to enable inspectors to check buried oil and gas pipe systems under high pressures [17]. Long et al. [220] investigated the propagation of GWs in steel bars embedded in soil and iron water pipes buried in soilation increase when mode phase velocity is higher than V_L_ in the soil (see Table 5 for details). Using the SAFE technique, Lowe and Castaings [221] studied arbitrary section elastic waveguide problems whereby GWs might leak into infinite surrounding solid materials. Hua [47] applied a hybrid SAFE method to buried pipes because a soil layer is a semi-finite space, unlike a pipe wall. Focusing parameters of a buried pipe, wave structures, dispersion curves and a buried coated pipe were calculated using the hybrid SAFE technique. To avoid energy reflection from the outer surface, an infinite element could be combined with finite elements (FEs) to simulate soil. Hence, a combination of the infinite element layer and the finite soil layer would properly perform the role of soil effect on GW propagation in buried pipes. Soil’s material properties differ from those of the viscoelastic coating materials of steel; thus, convergence analysis regarding element numbers used in finite and infinite soil layers and steel layer should be considered [47]. 

Rose et al. [222] studied phased array focusing inside buried and coated pipes. The findings showed that the energy of most GWs is attenuated in the various layers of soil. Theoretical analysis and GW propagation modelling were presented in buried and viscoelastic coated pipe configurations using the FEM technique. In addition, the SAFE technique was used to calculate the attenuation dispersion curve and phase velocity of coated pipes (see Table 5 for more details). The results indicated that GW mode selection with the smallest attenuation ratio plays an important role in producing improved penetration power in coated and buried pipe inspection. Leinov et al. [219] investigated the effects of sand conditions on pipes buried in sand. Such conditions included compacted and loose, water saturated and drained and under high applied pressure and mechanically compacted. They independently measured the attenuation for using L(0,2) and T (0,1) GW modes over a frequency range of 11 kHz to 34 kHz. T(0,1) mode exhibited higher attenuation values than L(0,2) mode for the physical condition of the sand. High pressure application on sand increased GW attenuation and modified its compaction. Similar attenuation values were observed in the sand with high applied pressure and mechanical compaction. Results showed that attenuation value increased in the drained sand and increased in the fully water-saturated sand to values comparable with those obtained from the compacted sand. This comparison showed that attenuation of torsional and longitudinal modes is governed by shear velocity in sand. Hence, an expected test range can be expected from the attenuation range that will enable reflections from defects to reflect back to the ring of transducers [219]. In fact, the large variability with sand conditions and the high values of attenuation revealed why test ranges of GWT are variable and are greatly decreased in buried pipes. Chiu et al. [223] showed that GW attenuation and buried depth were not related linearly. In fact, GW attenuation rate, which determines the range of detection, is mainly and permanently affected by soil pressure (see Table 5). When soil pressure was over 4700 kg/m^3^, the rate of attenuation tended to remain in a fixed range. Meanwhile, pipes of large size exhibit reduced GW attenuation. Under good conditions, anticorrosion tapes cause evident GW attenuation. 

Other investigations have concentrated on attenuative coatings. Rose and Barshinger [33] investigated GW propagation in an elastic hollow cylinder coated with a viscoelastic material. The multi-mode nature of GWs could obtain modes that would perform even in the presence of these coatings. To find these modes, a multilayer hollow cylinder model consisting of a viscoelastic layer was created using the global matrix method to express longitudinal propagation modes. This simulation resulted in an attenuation dispersion curve which revealed that low attenuation behaviour could be achieved by adjustment of the frequency and mode of operation. Moreover, higher-order modes could be achieved at high frequencies. In inspections, adoption of these modes provide an alternative to using only the lowest-order modes at very low frequencies [33]. Leinov et al. [224] studied the possibility of ultrasonic isolation of a buried FBE-coated pipe, using an added coating layer (PE foam coating layer) between the sand and the FBE-coated pipe. The impedance of this additional layer (PE foam coating layer) was smaller than that of the sand and the pipe because of its capability to tolerate excessive pressure and the load from the sand. Findings revealed that the measured values of GW attenuation in the buried FBE–PE foam-coated pipe were considerably smaller than those in the buried FBE-coated pipe for compacted and loose sand conditions. Significant reduction in GW attenuation regarding ultrasonically isolated buried pipe because of low impedance coating resulted in a considerable increase in the GW inspection range (see Table 5) [224]. Hua et al. [225] showed that a viscoelastic coating layer attenuated a large value of GW energy and therefore decreased the energy percentage leaking into the infinite media. This result revealed the potential of inspecting buried pipes by employing unburied-pipe-focusing parameters for thick and highly viscoelastic coated pipelines (see Table 5) [225]. Kwun et al. [17] stated that the magnitude of the torsional T(0,1) GW attenuation coefficient in coal tar enamel-coated buried pipes was two orders greater than that in aboveground bare pipes, thereby reducing the defect detection sensitivity and the inspection range (see Table 5). Kirby et al. [226] studied the RC of longitudinal and torsional GWs from defects in coated pipes. Recently, Nishino et al. [227] discussed attenuation characteristics of leaky T(0,1) mode propagation in a steel pipe thickly coated with petrolatum anticorrosion grease (see Table 5).

Lowe et al. [229] investigated GW propagation behaviour in 1 km (0.62 mi) sections of aboveground, immersed (in swamp) and buried (in soil) pipelines. For corrosion resistance, the buried pipe section was coated with bitumen, whereas in the lake region, it was coated by a layer of concrete outside the bitumen layer to improve corrosion resistance. A large range of aboveground section was inspected because the pipe had a large diameter with only a few features. However, in the immersed and buried sections, the test range was limited by attenuation and GW energy was absorbed in the protective concrete, bitumen layers and surrounding soil. On the one hand, usage of multiple frequencies in each section helped increase the test range from each test location [229]. On the other hand, inspecting over a frequency range is important because sensitivity to various type of defects varies with frequency in accordance with their dimensions, thus improving detection possibility [24]. This feature also improves the interpretation of reflections that come separately from defects of geometric features. Moreover, signal attenuation by different types of protective coatings can change considerably with frequency [230]. 

## 8. Concluding Remarks

This work attempted to present a general view of the detection, localisation and assessment of defects in pipes by using GW techniques. Clearly, an understanding of the effects of different types of defects on GW inspection results is significant. This work ultimately provided an overview of the interaction of GWs with axisymmetric defects, notch-like defects, cracks, corrosion and irregular and complex defects in cylindrical structures, such as pipes. The following paragraphs are intended to provide a brief overview of different types of GW transducers and the current GW techniques, such as GW focusing techniques. Currently, defect detection using GW technique is developed commercially to enable inspectors to identify a variety of defects and interpret the GW testing results. This work highlighted the simulation techniques for predicting GW characteristics and properties in elongated structures, with a focus on the FEM. As noted, the effects of controlling parameters, such as depth and circumferential and axial extents of a defect, on the RC, characteristics of GW propagation in aboveground and underground pipes, GW scattering and interaction with different types of defects, GW attenuation and effects of pipe features, pipe surface condition, surrounding medium and coating types of buried (e.g., in soil) pipes can be analysed numerically using 2D and/or 3D FEM models. These effects were also discussed. 

## Figures and Tables

**Table 1:** Detection of defects in pipes using GWs techniques.

No. (Defect Category)	Long Range GW Simulation Method and/or Experiment Technique		Study Purpose	Key Information	Long Range Guided Wave Type/Mode	Defect Type	See for Details
1 (Cracks) 2 (Corrosion)	A novel numerical procedure using a quantitative ultrasonic technique		To show wave scattering by circumferential cracks in steel pipes.	Wave function expansion was employed in the axial direction, and the problem was decomposed into anti-symmetry and symmetry problems. 3D wave-scattering problems were then reduced into two quasi-1D problems.	Torsional (T), flexural (F) and longitudinal (L) families	Planar circumferential cracks	Bai et al. [68]
	Axisymmetric and non-axisymmetric GW analysis		To show that the reflection coefficient (RC), from axisymmetric crack increases with frequency at a determined depth. The torsional waves were shown to be attractive for pipe inspection in practice.	The circumferential extent and depth were the controlling parameters of the reflection from cracks when the notch had a finite axial length; instead of a crack, reflections occurred at the end and start of the notch that led to a periodic variation in the RC dependent on the notch’s axial extent.	T(0,1), L(0,2), F(1,2), F(1,3), F(2,2)	Cracks and notches	Demma et al. [56]
	Experimental GW analysis (applicable to long-range tests)		To detect small axial cracks using experimental data	Axial cracks are generally undetectable because they have a small circumferential cross-sectional area (CSA) that can be negligible. Nevertheless, if the depth of axial cracks extends to approximately 70% of wall thickness, then the interaction mechanism between the torsional waves and the axial crack varies and the crack starts to produce a detectable signal along with the characteristic tailing signals.	T-waves	Axial cracks	Kwan et al. [69]
	A hybrid finite element (FE), method and modal representation technique		To investigate axisymmetric GW scattering by weldments of anisotropic bonding materials and cracks in steel pipes.	Resonant peaks of reflection coefficients at the cut-off frequencies of higher GW modes were observed when the frequency increased. As the length and slope of the crack increased, these peaks became increasingly pronounced.	Axisymmetric	Cracks	Zhuang et al. [54]
	Digital signal processing (DSP) algorithm technique and FEM		To locate, measure and characterise cracks accurately and systematically by quantifying the effects of scattering.	Auld’s formula for crack length modified to a reflected energy coefficient was used to measure cracks. The DSP technique significantly decreased the need for high-frequency signals to detect small cracks.	Circumferential		Valle et al. [57]
	3D numerical modelling		To provide insights into the use of circumferential SH GWs for quantitative testing of axial cracks by obtaining transmission and reflection coefficient curves.	A comparison was performed between dispersion equation and displacement wave structure from simulation to verify the efficiency of the FEM package.	Circumferential, shear horizontal (SH)	Axial crack defects (stress corrosion cracking, SCC)	Wang et al. [70]
	Different types of GW transducers	Synthetic focused guided wave (SFGW)	To show that focusing causes the reflection coefficient (RC) to approximately double relative to the sensitivity for unfocused fundamental T GW	CSM of SFGW allowed the application of focusing through the post-processing of previously collected data. The measured RC was dependent on the length of cracks for 100% and part of the depth of axial cracks at the frequency ranges using T families.	Torsional (T) family	Axially aligned defects (axial cracks)	Fletcher et al. [58]
		Common source method (CSM) transducer elements					
		Circumferential array of piezoelectric (applicable for long-range tests)	To estimate the defect depth using the defect image amplitude. To estimate the circumferential length using the complete width at half maximum of the image circumferential profile of the defect.	The synthetically focused system for each point of interest in the pipe wall was used for the recorded backscattered signals to create an image of the pipe’s reflecting features.	T(0,1)	Crack-like defects	Davis and Cawley [14]
		A shear horizontal guided wave magnetostrictive transducer	To detect axial cracks in pipes using circumferentially incident waves.	Damages could be estimated by comparing the signals calculated in uncracked pipes and cracked pipes.	Shear horizontal waves	Axial cracks	Woong Kim et al. [60]
		Transducer system (applicable for long-range tests)	To enable the defect localisation along the pipe length and present a rough estimation of defect sizing	A systematic analysis of frequency, guided wave mode, defect size and pipe size on the RC from notches was presented. The minimum and maximum values of the RC at different axial lengths were calculated and used for defect sizing.	T(0,1)	Corrosion axisymmetric defects	Demma et al. [24]
2 (Corrosion)	Phased array (PA) transducers	Time-delay periodic ring arrays (TDPRAs) as a novel GW transducer model by FEM calculation	To generate axisymmetric GWs in hollow cylinders	GWs reflected by non-axisymmetric and axisymmetric corrosion defects were analysed, and 3D and 2D FE simulations were employed.	L(0,1) and L(0,2)	Non-axisymmetric and axisymmetric corrosion defects	Zhu [5]
	Phased array (PA) transducers	Higher-order mode cluster (HOMC)-GW, using linear phased array (PA) transducers and conventional circular transducers	To show the capability of GWs in obtaining improved imaging of small defects through the pipe cross section (CS) using high frequencies.	High-frequency modes were investigated to identify and size defects.	Circumferential GWs	Pinhole-like defects (corrosion) and axial notches (axial cracks)	Satyarnarayn et al. [8]
							Balasubramaniam et al. [9]
		Circumferentially distributed PA used for focusing technique	To achieve excellent circumferential, axial location and detection of defects. Defect circumferential sizing can be done by performing circumferential scans at defect distances.	GW energy could be focused successfully on any location in a pipe by applying varying time-delay inputs and amplitudes to the PA. GW focusing technique enhanced circumferential resolution and penetration power.	T(m,1) and L(m,2)	Volumetric elliptical corrosion, volumetric through-wall hole, planar saw cut defects	Mu et al. [71]
	Piezoelectric transducers (PZT)	Piezoelectric crystal-based transducers/2D FE model using Abaqus software	To detect defects with diameter as small as 1.5 mm and 25% penetration wall thickness by using the system.	A manual pipe crawler with a supply for holding the wedge and important hardware, such as the encoder and data acquisition card, were used.	Higher-order circumferential G	Pitting corrosion between the pipe and pipe support	Shivaraj et al. [13]
		Piezoelectric transducers (PZT) and FE simulation (applicable for long-range tests)	To show the complexity of the results of the reflection signals of defects received from the overlap amongst the reflections of the defects’ edges with varying features.	The reflection of GWs received from the back and front edges of defects in pipelines was reported. A new strategy for the defects’ quantitative characterisation was proposed.	L(0,1), L(0,2) F(1,2), F(1,1), F(1,3)	Notch-type defects, corrosion	Wang et al. [72]
	FE 3D analysis of the RC		To simulate defect clusters and multiple part-depth defects using the superposition approach.	The RC of the T(0,1) mode was a function of the axial separation and frequency and was independent of circumferential position.	T(0,1)	Multiple circular holes/localised corrosion	Løvstad and Cawley [73]
	FE analysis by Abaqus software		To identify the presence of sharp edges in corrosion patches according to the RC spectrum analysis.	The superposition of high and low spatial frequency contents of defects provided a good approximation to the RC from the full profiles.	T(0,1)	Sharp edges in corrosion profiles	Cawley et al. [74]
	Reconstruction algorithm for the probabilistic inspection of damage (RAPID)	Leave-in place sensors (an array of 16 low-cost transducers)	To create tomographic images of multiple defects. Defects could be detected by employing sparse array.	The defect severity and location were accurately predicted by using the RAPID algorithm employing multiple frequency data sets.	GW mode	Corrosion and pitting defects	Velsor et al. [75]
3 (Other defects)		Nonlinear guided wave and tomography RAPID	To detect, locate and image micro-defects in pipe structures.	Nonlinear tomography was used to provide the micro-defect images in pipe structures.	Nonlinear GWs	Thermal fatigue damage	Cho et al. [76]
	Non-contact shear horizontal (SH) wave EMAT and piezoelectric lamb wave transducers	Boundary element method (BEM) and normal mode expansion (NME)	To conduct research on the sizing potential of 2D-shaped defects in a structure. At low frequency, the SH and lamb waves were shown to be insensitive to the stringer internal inclusions.	The energy redistribution to the higher-order wave modes that occurred as a monotonic trend did not hold generally at a high frequency.	Lamb and shear horizontal (SH) waves	Axisymmetric defects with varying depth profiles (surface breaking half-elliptical-shaped defects)	Rose et al. [77]
	Electromagnetic acoustic transducers (EMAT)	Tomographic reconstruction technique	To propose a novel method for suppressing the unwanted S_0_ mode according to the Poisson effects of materials through optimising the inclination angle of the equal transduction force of the EMAT employed for detection and generation.	The potential advantage of enhancing the frequency of inspection was known as constant group velocity (CGV) point, where the group velocity remained constant over a broad range of wall thickness differences. The phase velocity allowed the evaluation of accurate wall thickness from phase angle calculations.	A_0_ and S_0_ mode	Corrosion, erosion	Nagy et al. [78]
	Defect imaging technique		To identify pipe ends and holes in the images obtained by a defect imaging technique.	A defect imaging technique was used to overcome the GW complexities.	Shear horizontal vibration and torsional modes	Surface breaking half-elliptical shape defects	Hayashi et al. [50]
	Thickness shear mode piezoelectric transducers		To show that torsional modes are preferable over longitudinal modes for detecting various defects in pipes.	Different defects (circumferential and longitudinal defects) could be inspected using T(0,1) mode. However, some errors were found in the area behind the defects where GWs did not propagate or reflect back to the transducer	T(0,1)	Longitudinal and circumferential defects	Liu et al. [79]
	Different types of focusing techniques	Focusing inspection technique (applicable for long-range tests)	(1) This measurement technique operated efficiently with volumetric through-wall hole and planar saw cut defects. (2) To report that different shaped defects with the same CSA show different reflections.	Circumferential extent, depth and location of various shaped defects were studied by focusing on 44 circumferential locations around the pipes at a particular distance and the maximum amplitude of the defect signal obtained for each circumferential focal position. Theoretical calculations and experimental results were compared to measure the circumferential lengths of the defects.	T(0,1)	Volumetric spherical shape corrosion, volumetric through-wall hole, planar saw cut	Mu et al. [80]
		PA focusing technique and 3D FEM simulation	To improve the results obtained by using UGW inspection by concentrating the energy onto defects and to show that the PA focal location is rarely affected by a limited number of welds.	Focusing could enhance the energy impinging onto the defect, decrease false alarm ratio, increase the distance of GW propagation and locate the defect. The amplitude factors and time delay for GW array focusing were nonlinear functions of the active frequency, excitation condition, pipe size and focal distance.	T(m,1) family	Defects beyond weld	Rose et al. [81]
		(1) Signal-based focusing (SBF) and (2) angular profile tuning (APT)	To discuss and compare two focusing techniques that can increase defect detectability by focusing the UGWs by transducer arrays	(1) SBF focused UGWs by employing cross-correlation analysis. (2) The APT approach depended on a theoretical prediction of the pressure field of UGWs made by an ultrasonic transducer attached to the pipes.	Non-axisymmetric GWs, F(1,m) and F(2,n)	Through-wall hole, circumferential notches	Kang et al. [15]
	Semi analytical method	Experimental and FEM superposition approaches	To reconstruct the RC of a tapered defect by employing the transmission and reflection characteristics of tapered down and up steps. The RC from tapered notches showed a similar form when received from the tapered notch with an average extent.	At high frequencies and at a given taper angle, the tapered defects could not be detected easily because the amplitude of the RC peaks decreased whilst the frequency increased. Inhomogeneous modes of down-step V-notches interacted with those of the up-step ones, causing the RC from V-notches to be lower than that from the tapered defects of the same depth.	T(0,1)	Surface defects	Cawley et al. [82]
	FE analysis of 3D scattering of guided waves.		To investigate the 3D scattering of GWs by a through-thickness cavity with an arbitrary shape.	The mode amplitude was measured by writing the nullity of the full stress at the cavity boundary. The results were compared with a solution measured with FE method-efficient models, which allowed very fine meshes to be defined for accurate scattering modelling.	SH and lamb waves (non-propagating and propagating)	Arbitrary shapes: (1) Elliptical (Clusters) and (2) irregular shaped defects	L. Moreau et al. [83]
	GW FE modelling		To propose a procedure for optimising the measurement of the scattering matrix of irregular defects using FEM.	The proposed procedure was in accordance with the combination of effective FE model formulation and the following parameter optimisation: number of incident angles in the scattering matrix, smoothness of defect geometry, size of the absorbing region and element size.	SH_0_ S_0_ A_0_	Irregular defects/corrosion defect profile	L. Moreau et al. [84]
	FE 3D analysis using RC		To investigate the effects of complex defect profiles on the RC from 3D defects in pipes. The analyses presented a practical approach to determining the maximum depth of complex defects from the RC behaviour.	At a provided maximum depth of a finite defect, the RC peak from defects was a linear function of the circumferential length of the defects and was independent of their shapes.	T(0,1)	Complex-shaped defects	Cawley et al. [85]

**Table 2:** Selection of parameters for FE models based on the parameter formula description.

**Signal impulse time**	τ=n/f	With such a signal, the force function energy can efficiently concentrate throughout a finite interval in the frequency and time domains; *n* expresses the number of pulse cycles, τ is the signal impulse time, and *f* is the central frequency.
**Signal time step (time increment size)**	$\Delta t<\frac{0.8L_{\max}}{V_{g}}$	If the travel distance of the fastest wave mode within a time step follows this condition, then the propagation of guided waves (GWs) would be within an element. In this way, an accurate calculation and analysis of the structure can be ensured. By contrast, the solution could be unpredictable and diverge rapidly if the increment is large. The group velocity *V_g_* expresses the fastest GWs at the determined frequency. Frequency is selected at the beginning of the simulation in accordance with the dispersion curves. *L*_max_ is the element length.
**Total time period**	$T>\frac{2L}{V_{g}}$	This condition ensures that the transducer arrays can receive at least one reflected signal from the pipe end when employing pulse-echo signals to identify the pipe during the simulation. *T* is the total time of the simulation. *L* is the pipe length.
**Element size**	$L_{\max}\leq \frac{\lambda_{\min}}{15}$	For good convergence, the maximal element size (*L*_max_) or the spatial sampling interval must be sufficiently small to enable the identification of the smallest wavelength in the computation domain. *λ*_min_ is the smallest wavelength. *L*_max_ is the maximal element size.
**Frequency**	*f*	The excitation frequency is selected at the beginning of the simulation in accordance with the dispersion curves.

**Table 3:** Defect classification follow-up priority, F, scheme defined as the product of the defect category, C, and directionality distribution, D. If F = 0, then a classification is assigned for the weld feature; if F = 1, then a low priority is assigned for follow-up (yellow-shaded cell); if F = 2, then a medium follow-up priority is required (orange-shaded cell); if F ≥ 3, then a high follow-up priority is required for signals from defects (red-shaded cell) [112,185].

Follow-up Priority F = C × D		Defect Category, C		
		1	2	3
Directionality distribution (D)	0	0	0	0
	1	1	2	3
	2	2	4	6
	3	3	6	9

**Table 4:** Factors affecting guided wave system operation [178].

Difficulty Degree		Condition of Surface	Geometry	Pipe Content
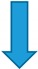 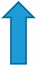	Difficult		Numerous bends	
		Concrete coating	Branches	Waxy or sludgy deposits
		Bitumastic coating		
		Plastic coating		
		Heavy pitting		High-viscosity liquid
		Light pitting		
		Fusion-bonded epoxy		Low-viscosity liquid
		Mineral wool insulation		
		Smooth well-bonded paint	Straight length	Gas
	Easy	Bare metal		

**Table 5:** Measured values for GWs propagation in different buried/coated pipe conditions.

Waveguide Condition	Surrounding Medium and Coating Type		Density (kg m^−3^)		Velocity		Attenuation (dB)		Test Range (m)	Content	Frequency (kHz)	Measurement Technique/Instruments	Ref
					V_L_ (ms^−1^)	V_S_ (ms^−1^)	T(0,1)	L(0,2)					
**Coated and buried (in soil with different depths [D]) steel pipes (two different diameters)**	Coal tar enamel-coated and soil	D = 0 m	N/A		N/A	3250	0.17 ± 0.01	N/A	2-8 at D = 1.7 m	Moisture	5	MsS instrument system for experiment tests	[17]
		D = 0.5 m					0.89 ± 0.03				10		
		D = 1.1 m					1.72 ± 0.20				20		
		D = 1.7 m					3.56 ± 0.53				30		
**Ductile iron buried pipe buried**	Clay and crushed concrete	Water	1000		1480	0		L(0,1), F(1,1)	1–10	Water	1–5	On-site GW inspection system	[220]
		Ductile iron pipe	7100		5500	3050							
		Saturated soil	1000		1500	25–100							
		Unsaturated soil	1900		250–1250	100							
**Buried pipe**	Loose sand		1455		500	120	T(0,1)		15–30	N/A	10–35	Experiment with commercial transducer rings (Wavemaker G4 instrument) and disperse modelling software for simulation	[224]
	Compacted sand		1724										
**Fusion-bonded epoxy (FBE)-coated pipe**	Loose sand		1485										
	Compacted sand		1635										
**polyethylene (PE) foam–FBE-coated pipe**	Loose sand		1475										
	Compacted sand		1630										
**Coated and buried steel pipe**	Steel hollow cylinder		7860		5850	3230	T(m,n)	L(m,n)	N/A	Hollow cylinder pipe	100–500	Hybrid SAFE	[47]
	Bitumastic 50		1200		1900	750							
	Unsaturated soil		1900		1000	100							
Viscoelastic coated and buried steel pipe	Enamel paint		1000		1680	770	(1) −0.65 (2) −1.26 (3) −15.4 (4) −22	(1) −0.62 (2) −0.96	N/A	Hollow pipe	50	(1) SAFE and (2) FEM (3) FEM for buried pipe in stiff clay (4) FEM for buried pipe in dense sand	[222]
	Bitumastic 300 M		600		1500	680	(1) −0.33 (2) −0.44 (3) −17.1 (4) −23.1	(1) −0.31 (2) −0.44					
	Bitumen tape		1200		1900	860	(1) −8.62 (2) −10.65 (3) −27.1 (4) −28.1	(1) −8.09 (2) −8.65					
	Single-layer FBE		800		1900	860	(1) −0.44 (2) −0.63 (3) −12.6 (4) −19	(1) −0.4 (2) −0.26					
	Bitumastic 50		1200		1900	750	(1) −1.72 (2) −2.92 (3) −17 (4) −23.9	(1) −1.79 (2) −2.73					
	Stiff clay		1730		157	64.3	N/A	N/A					
	Dense sand		1840		209	100	N/A	N/A					
Pipe buried in sand (infinite soil medium)	Loose sand		1455		400	83	2.2~2.5	1.5~1.6	N/A	Hollow cylinder pipe	11–34	Transducer rings (Wavemaker G4 instrument system) for experiment tests and data interpretation using disperse modelling software	[219]
	Compacted sand		1620		400	118	3.5~4.6	2.5~2.6					
	Mechanically compacted sand		1660		600	122	3.5~5.5	2.1~3.1					
	Sand under overburden pressure		1895		400	44	1.6~2	1~1.1					
	Water-saturated sand		1895		400	77	2.7~3.2	1.85~1.90					
	Drained sand		1724		400	127	3.1~5.1	2~2.9					
Buried pipes (with different diameters) in soil (underground within the level of −2~−3.5 m in depth)	PE-coated pipes) (three pipe diameters [D])		D1 = 88.4 mm	N/A	N/A	N/A	−29~−42	N/A	<2	Water	34–48	On-site GW inspection using Wavemaker G3 instrument system (experiment tests)	[223]
			D2 = 115.6 mm	N/A	N/A	N/A	−13~−19	N/A			29–45		
			D3 = 168.7 mm	N/A	N/A	N/A	−3~−7	N/A			19–30		
	Bitumen-coated pipes (three pipe diameters [D])		D1 = 88.4 mm	N/A	N/A	N/A	−27~−48	N/A			34–48		
			D2 = 115.6 mm	N/A	N/A	N/A	-21~-30	N/A			29–45		
			D3 = 168.7 mm	N/A	N/A	N/A	−2~−12	N/A			19–30		
4 mm viscoelastic coated and welded buried pipe in infinite soil	Fibrous coal tar coating		−1 °C	2500	1000	2300	Longitudinal attenuation factor: 0.011	Shear attenuation factor 0.1	12.2	Hollow cylinder	25, 30 and 35	SAFE technique for coated and buried pipes using hybrid focusing technique	[225]
	Fibrous coal tar coating		21 °C	2500	800	2100	Longitudinal attenuation factor: 0.029	Shear attenuation factor 0.8					
Coated pipes (different wall-thicknesses [WT] and temperatures)	Petrolatum anticorrosion grease (PAG)		20 °C	N/A	N/A	78~277	8~10	N/A	3	Hollow cylinder	30	Piezoelectric ring-shaped sensor system for experiment	[227]
			30 °C				4~6				40		
			40 °C				2~3				50		
			WT = 4 mm	N/A	N/A	78~277	3~10	N/A			30		
			WT = 5 mm				3~9				40		
			WT = 6.5 mm				2~7.5				50		
Coated pipe	Bitumen		28 °C	970	N/A	460	4.046–4.668 db/m		7.53	Hollow pipe	Frequency regime: 0.2–3	Disperse modelling software and transducer arrays for experiment	[228]
	PE			950	N/A	950	2.235–2.453 db/m		14				
	Mineral wool			2500	N/A	2000	Near zero		N/A				
